# Genome-wide comparative analysis of the HSP90 gene family in four Ipomoea species and functional insights into the potentiality of IbHSP90–2 in low temperature tolerance

**DOI:** 10.3389/fpls.2026.1791008

**Published:** 2026-03-06

**Authors:** Lizhe Shu, Kun Zhu, Xingyu Wang, Lilin Cheng, Shuo Zhou, Mingku Zhu, Xiaowan Gou, Zongyun Li

**Affiliations:** 1 The Key Laboratory of Biotechnology for Medicinal and Edible Plants of Jiangsu Province, School of Life Sciences, Jiangsu Normal University, Xuzhou, China; 2 Institute of Integrative Plant Biology, School of Life Sciences, Jiangsu Normal University, Xuzhou, Jiangsu, China

**Keywords:** abiotic stress, HSP90, *I. cordatotriloba*, *I. triloba*, sweetpotato, *I. trifida*, cold tolerance

## Abstract

**Introduction:**

The 90-kDa heat shock protein (HSP90) acts as an essential molecular chaperone, involving plantresilience against diverse abiotic and biotic stresses. Although *HSP90* genes have been studied in various plant species,they have not yet been characterized in sweetpotato.

**Methods:**

In this study, we performed genome-wide identification, chromosomal localization, phylogenetic analysis, collinearity analysis, and promoter analysis of HSP90 genes in *I. batatas* and three closely related *Ipomoea* species. We also investigated gene expression patterns under cold, salt, and PEG-induced drought stresses, constructed protein-protein interaction networks, and determined the subcellular localization and functional role of *IbHSP90-2* via heterologous expression in yeast.

**Results:**

We identified 10 *HSP90* genes in *I. batatas*, 11 in *I. trifida*, and 10 each in *I.triloba* and *I.cordatotriloba*. Chromosomal localization analysis showed an uneven distribution of *HSP90* genes across 15 chromosomes. Phylogenetic and collinearity analyses grouped these genes into four subfamilies,with segmental duplication being the main factor for gene family expansion. Promoter analysis revealed multiple stress-responsive cis-acting elements, indicating that *IbHSP90* genes may play a role in stress regulation. Expression analysis revealed that most IbHSP90s were downregulated under cold, salt, and PEG-induced drought stresses. Additionally, we developed a protein -protein interaction network that identified connections with heat shock factors. Experiments on subcellular localization revealed that IbHSP90-2 is present in both the cytoplasm and nucleus. Finally, heterologous expression of *IbHSP90-2* in yeast compromised cold resistance.

**Discussion:**

This study offers a significant resource for comprehending the evolution of plant HSP90 proteins and supports the genetic enhancement of cold tolerance in sweetpotato.

## 1 Introduction

Heat shock proteins (HSPs) are molecular chaperones found across animals, plants, and microorganisms (Ren et al., 2026). They assist in proper protein folding and repair of damaged protein structures, thereby helping plants cope with abiotic stress and maintain normal physiological functions (Picard, 2002; Pratt and Toft, 2003, Sangster and Queitsch, 2005, Wang et al., 2019). The five families of HSPs, categorized by molecular weight and sequence homology, include HSP100, HSP90, HSP70, HSP60, and sHSP (Morimoto, 1998). HSP90 proteins, weighing around 90 kDa, function as molecular chaperones involved in plant stress responses by modulating protein folding (Johnson and Brown, 2009, Driedonks et al., 2015). HSP90 proteins have been discovered in various plants, including *Arabidopsis thaliana* (Milioni and Hatzopoulos, 1997), *Nicotiana tabacum* (Song et al., 2019), *Hordeum vulgare* (Chaudhary et al., 2019), *Avena sativa* (Peng et al., 2024), *Olea europaea subsp. Europaea* (Bettaieb et al., 2020), *Dendrobium officinale* (Wang et al., 2022), *Gossypium hirsutum* L (Hao et al., 2025), *Populus* (Zhang et al., 2013), *Zingiber officinale Roscoe* (Xiao et al., 2025) and *Solanum tuberosum* (Li et al., 2020). HSP90 proteins typically comprise three distinct domains: ATP binding and hydrolysis sites at the N-terminus, a central M region, and a dimerization-functional C-terminus (Prodromou and Pearl, 2003). HSP90s fall into four classes (chloroplast: CP, mitochondrial: MT, endoplasmic reticulum: ER, and cytoplasmic: Cyt) according to their subcellular localization, phylogenetic relationships and expression patterns (Milioni and Hatzopoulos, 1997).

Research has shown that HSP90 protein is involved in a series of stress responses in plants, including temperature, salinity, drought, and pathogen infection. For instance, in *A.thaliana*, the autophagy receptor protein NBR1 interacts with ROF and HSP90.1, mediating their selective autophagic degradation to clear heat stress memory (Thirumalaikumar et al., 2021). Overexpression of *DcHsp90–6* in *A.thaliana* significantly enhances thermotolerance in transgenic seedlings (Xue et al., 2023). Similarly, in alfalfa, four HSP90 genes are involved in cold stress response, while six HSP90 genes participate in the cold stress response of cabbage (Sajad et al., 2022; Liu et al., 2025). In contrast, overexpression of *AtHsp90.2*, *AtHsp90.5*, or *AtHsp90.7* increases plant sensitivity to salt and drought stresses (Song et al., 2009). Conducted a genome-wide identification of the HSP90 gene family in Rosa chinensis and performed qRT-PCR analysis, which revealed that *RcHSP90-1-1*, *RcHSP90-5-1*, and *RcHSP90-6–1* are crucial regulators of salt and drought stress responses in the Sweet Avalanche and Wang Xifeng varieties (Xu et al., 2024). The HSP90 gene family also plays a crucial role in cotton salt stress response (Hao et al., 2025). Notably, HSP90 proteins are essential for drought stress responses in various plant species. In cassava, HSP90.9 positively regulates drought resistance by promoting ABA biosynthesis through MeWRKY20 and enhancing antioxidant activity to scavenge H_2_O_2_ (Wei et al., 2020). Beyond abiotic stress responses, HSP90 also plays a vital role in plant defense against pathogen infections. For example, cassava HSP90 exerts a dual regulatory role against *Xanthomonas phaseoli* by interacting with CPK1 and XopC2 (Wei et al., 2024). Besides its roles in stress responses and pathogen defense, HSP90 is also involved in plant root development. In *A.thaliana*, HSP90 regulates root growth by modulating the polar distribution of PIN1 (Samakovli et al., 2021). Similarly, overexpression of *SlHSP90.2* in tomatoes leads to changes in root biomass and architecture (Yadav et al., 2021).

Sweetpotato serves as a significant food crop and a high-quality resource for both feed and industrial applications (Yan et al., 2022). Owing to its strong adaptability, broad cultivation distribution, superior yield, and exceptional nutritional value, sweetpotato has been widely grown throughout China for many years (Sun et al., 2014; Liu et al., 2020; Meng et al., 2024). Nevertheless, constraints in arable land availability have limited its planting area to only about 3% of the total farmland substantially less than that of wheat, maize and rice. Furthermore, soil salinization resulting from industrial pollution, excessive use of fertilizers and pesticides, along with extreme climatic events, continues to adversely affect sweetpotato production and quality (Chen et al., 2017; Jia et al., 2024). The genome sequencing and assembly for the hexaploid sweetpotato variety Taizhong 6 and its three diploid relatives, *I. trifida* (NCNSP0306), *I. triloba* (NCNSP0323), and *I. cordatotriloba* (xiaoshu) have been completed. It has become feasible to systematically identify and analyze key gene families across the sweetpotato genome (Yang et al., 2017; Wu et al., 2018; Xiao et al., 2025). This progress provides a foundation for enhancing both the yield and quality of sweetpotato through genomic approaches.

This study characterizes the *HSP90* gene family in *I. batatas* and its three diploid relatives. After classifying the identified *HSP90* genes into four subfamilies, we conducted extensive analyses of their protein physicochemical properties, chromosomal localization, phylogenetic relationships, gene structure, promoter cis-elements, protein-protein interaction networks, and expression profiles. This study offers valuable understanding of the evolutionary dynamics and functional roles of HSP90s in sweetpotato, laying a theoretical groundwork for enhancing stress tolerance, yield and quality in sweetpotato.

## 2 Materials and methods

### 2.1 Plant materials

All experiments were conducted using the sweetpotato cultivar Xuzishu 8 (XZ8) as the sole experimental material. XZ8 is an elite and widely cultivated purple sweetpotato variety that exhibits multiple superior agronomic traits, including high yield, high anthocyanin content, early maturity, and excellent edible quality. Notably, it is a versatile cultivar applicable to both fresh consumption and industrial processing, with considerable application potential (Song et al., 2024). However, low-temperature stress acts as a critical limiting factor that constrains its geographical distribution, large-scale cultivation, and yield stability. For subcellular localization assays, *N. benthamiana* plants were cultivated at Jiangsu Normal University.

### 2.2 Identification of *HSP90* genes in *Ipomoea* species

Sweetpotato data was sourced from the Ipomoea Genome Hub (https://sweetpotato.com/) (Yan et al., 2024). Data for *I. trifida* and *I. triloba* were acquired from the Sweetpotato Genomics Resource (https://sweetpotato.uga.edu/) (Wu et al., 2025). Data related to *I. cordatotriloba* was obtained from the National Genomics Data Center (https://ngdc.cncb.ac.cn/PRJCA027907) (Xiao et al., 2025). BLAST analyses were conducted on four *Ipomoea* species with seven HSP90 protein sequences of *A.thaliana* as queries (Milioni and Hatzopoulos, 1997). The HMM profile for the HSP90 DNA-binding domain (PF00183) from Pfam (http://pfam.xfam.org/) was utilized to identify proteins with conserved HSP90 domains via the Advanced HMMER Search program in TBtools (Chen et al., 2020; Mistry et al., 2021; Chen et al., 2023). All putative protein sequences were additionally confirmed to contain the HSP90 domain via SMART, Pfam and NCBI CD-search. The physicochemical properties of identified HSP90 members were analyzed using Expasy’s ProtParam tool(https://web.expasy.org/protparam/), and their subcellular localization was predicted with Plant-mPLoc (Gasteiger et al., 2003; Chou and Shen, 2010).

### 2.3 Chromosome distribution and phylogenetic analysis of *Ipomoea* HSP90s

Using TBtools and genome annotation files, the chromosomal locations of the identified HSP90 genes in *Ipomoea* species were mapped. A maximum-likelihood phylogenetic tree was generated using MEGA11 software from the aligned HSP90 protein sequences. The tree file was uploaded to the Interactive Tree of Life (iTOL) platform for visualization and graphical adjustments (Letunic and Bork, 2021, Tamura et al., 2021).

### 2.4 Protein conserved motif and gene structure analyses of *Ipomoea* HSP90s

Conserved motifs of HSP90s were identified via MEME Suite (https://meme-suite.org/meme/tools/meme) (Bailey et al., 2009). Gene structural features were extracted, and their diagrams generated in TBtools. Finally, TBtools was used to integrate and visualize the gene structures and conserved motifs of HSP90s across *Ipomoea* species (Chen et al., 2020, Chen et al., 2023).

### 2.5 Synteny analysis and calculation of Ka/Ks values of *Ipomoea* HSP90s

The synteny of the four Ipomoea genomes was assessed and visualized using TBtools, which also facilitated the calculation of Ka, Ks, and Ka/Ks ratios for duplicated HSP90s (Chen et al., 2020, Chen et al., 2023).

### 2.6 Cis-regulatory element analysis

The 2.0 kb upstream sequences of the start codon for all family members were obtained from the sweetpotato genome annotation. The PlantCARE online database to predict cis-regulatory elements in the promoter sequences (Lescot et al., 2002).

### 2.7 Analyses of expression patterns and quantitative real-time PCR

*IbHSP90s* data from diverse tissues and abiotic stress conditions (high, low temperature, salt, drought) were obtained from the Sequence Read Archive using accession numbers PRJNA744414, PRJNA486421, PRJNA987163, PRJNA1209623, PRJNA631585, and PRJNA917061. Data for *ItfHSP90s* and *ItbHSP90s* were sourced from the Sweetpotato Genomics Resource. Uniformly grown XZ8 seedlings were used for all treatments. For stress treatments, 4-week-old *in vitro*-cultured seedlings were subjected to drought and salt stress with 20% (w/v) PEG6000 and 200 mM NaCl, respectively. Additionally, 4-week-old seedlings after germination were transplanted into nutrient soil and cultured for one week to ensure vigorous growth, followed by low-temperature stress at 4 °C. For all stress treatments, fully expanded leaves from the same nodal position were harvested and pooled as mixed samples at 0, 3, 6, 12, 24, and 48 h post-treatment. For foliar hormone treatments, 4-week-old uniformly grown XZ8 seedlings with consistent vigor were selected and separately sprayed with 100 μmol/L jasmonic acid (JA), abscisic acid (ABA), salicylic acid (SA), indole-3-acetic acid (IAA), gibberellin (GA), and melatonin (MT). After 12 h of hormone treatment, leaves from the same nodal position were harvested and pooled as mixed samples. All collected samples from both stress and hormone treatments were immediately frozen in liquid nitrogen and stored at −80 °C until total RNA extraction. Three biological replicates were conducted independently, each comprising three plants. *IbHSP90* transcript levels were quantified via qRT-PCR, using *IbARF* as the reference gene (Moraes et al., 2025). Gene expression was measured using the 2-ΔΔCT comparative method.

### 2.8 Protein-protein interaction and functional enrichment analyses of IbHSP90s

The protein-protein interaction networks of IbHSP90 were predicted using the STRING database (https://string-db.org/) with a maximum of 10 interactors and a high confidence score of 0.7 (Von Mering et al., 2005). The prediction was based on their *A.thaliana* homologs identified through sequence alignment. Functional enrichment analysis of the 10 IbHSP90s was conducted using TBtools (Chen et al., 2020, Chen et al., 2023).

### 2.9 Subcellular localization

The fusion vector p1300-NLS-Red-GFP-IbHSP90–2 was created by inserting the complete CDS of *IbHSP90–2* into the p1300-NLS-Red-GFP vector, followed by transformation into *Agrobacterium* GV3101. Bacterial suspensions were cultured and infiltrated into *N. benthamiana* leaves. After 48 hours post-infiltration, leaf segments were examined using a confocal laser-scanning microscope. By comparing GFP fluorescence of the fusion construct with the NLS signal, the subcellular localization of IbHSP90–2 was confirmed.

### 2.10 Assays for assessing the tolerance of yeast transformants to low temperatures.

The cold tolerance of transgenic yeast was assessed as previously described (Xiang et al., 2020). INVSc1 (pYES2-IbHSP90-2) and INVSc1 (pYES2) cultures were grown separately in SG-Ura liquid medium at 28°C with shaking at 200 rpm for 36 hours. Following centrifugation at 5000 rpm for 10 minutes, the cell density was standardized to an OD600 of 0.8 using SG-Ura medium. Samples underwent 72 hours of cold stress at either 4°C or -20°C, followed by serial dilution. Subsequently, 5 μL aliquots were spotted onto SG-Ura plates. Plates were incubated at 28°C to assess yeast growth. The samples treated at -20°C and 4°C for 0, 3, 6, 9, 12, 24, 48, and 72 h were separately cultured at 28°C for 12 h, and then the cell densities (OD600) were measured.

## 3 Results

### 3.1 Identification and characterization of *HSP90* family members in four *Ipomoea* species

Genome analysis identified 10 *HSP90* genes in *I. batatas*, 11 in *I. trifida* and 10 each in *I. triloba* and *I. cordatotriloba*. Analysis of the physicochemical properties of HSP90 proteins from the four *Ipomoea* species revealed consistent characteristics (**Supplementary Figure 1**). Amino acid lengths varied between 296 and 816 residues, averaging 624 residues (**Supplementary Figure 1a**). Correspondingly, the molecular weights (MW) varied from 32.58 kDa to 93.63 kDa. IbHSP90–6 had the highest MW (93.63 kDa), while ItbHSP90–4 was both the shortest (296 aa) and lightest (32.58 kDa) protein (**Supplementary Figure 1b**). All proteins were acidic, with isoelectric points (pI) between 4.44 (ItbHSP90-9) and 5.63 (IbHSP90-6) (**Supplementary Figure 1c**). Based on the instability index, approximately 90.24% (37 of 41) of the proteins were predicted to be stable (index < 40), with the remaining four classified as unstable (**Supplementary Figure 1d**). The aliphatic index ranged from 79.22 (ItfHSP90-9) to 90.65 (ItbHSP90-3) (**Supplementary Figure 1e**). All members showed a Grand average of hydropathicity (GRAVY) score exceeding -0.1, reflecting a predominantly hydrophobic nature (**Supplementary Figure 1f**). Subcellular localization predictions suggested that most HSP90s target the endoplasmic reticulum or cytoplasm, with a minority localized to mitochondria or the nucleus (**Supplementary Table 1**).

### 3.2 Chromosomal localization and phylogenetic analysis of *Ipomoea* HSP90s

**Figure 1:**
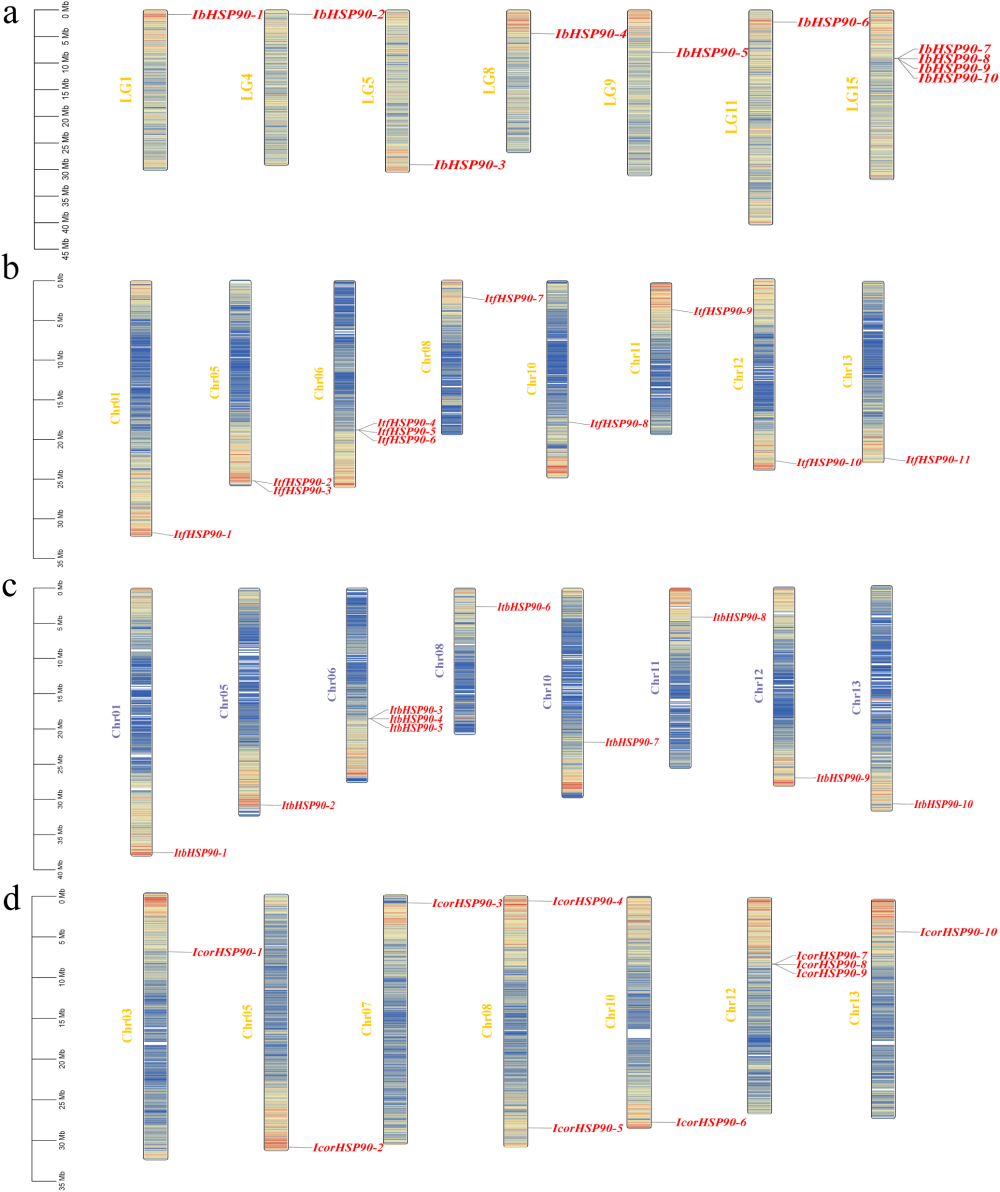
Chromosomal localization and distribution of *IbHSP90s* **(a)**, *ItfHSP90s* **(b)**, *ItbHSP90s* **(c)** and *IcorHSP90s* **(d)**. The bars represented chromosomes, the chromosome numbers were displayed on the left side, and the gene names were displayed on the right side.

The chromosomal distribution of *HSP90s* across the four *Ipomoea* species was uneven (**Figure 1**). In *I. batatas*, chromosome 15 exhibited the highest concentration, containing four *HSP90* genes. In the diploid relatives, *HSP90* genes were identified on chromosome 6 of *I. trifida*, chromosome 6 of *I. triloba*, and chromosome 12 of *I. cordatotriloba*. The remaining chromosomes contained either one or no *HSP90* genes. Based on their chromosomal positions, all identified *HSP90* genes were systematically renamed.

**Figure 2:**
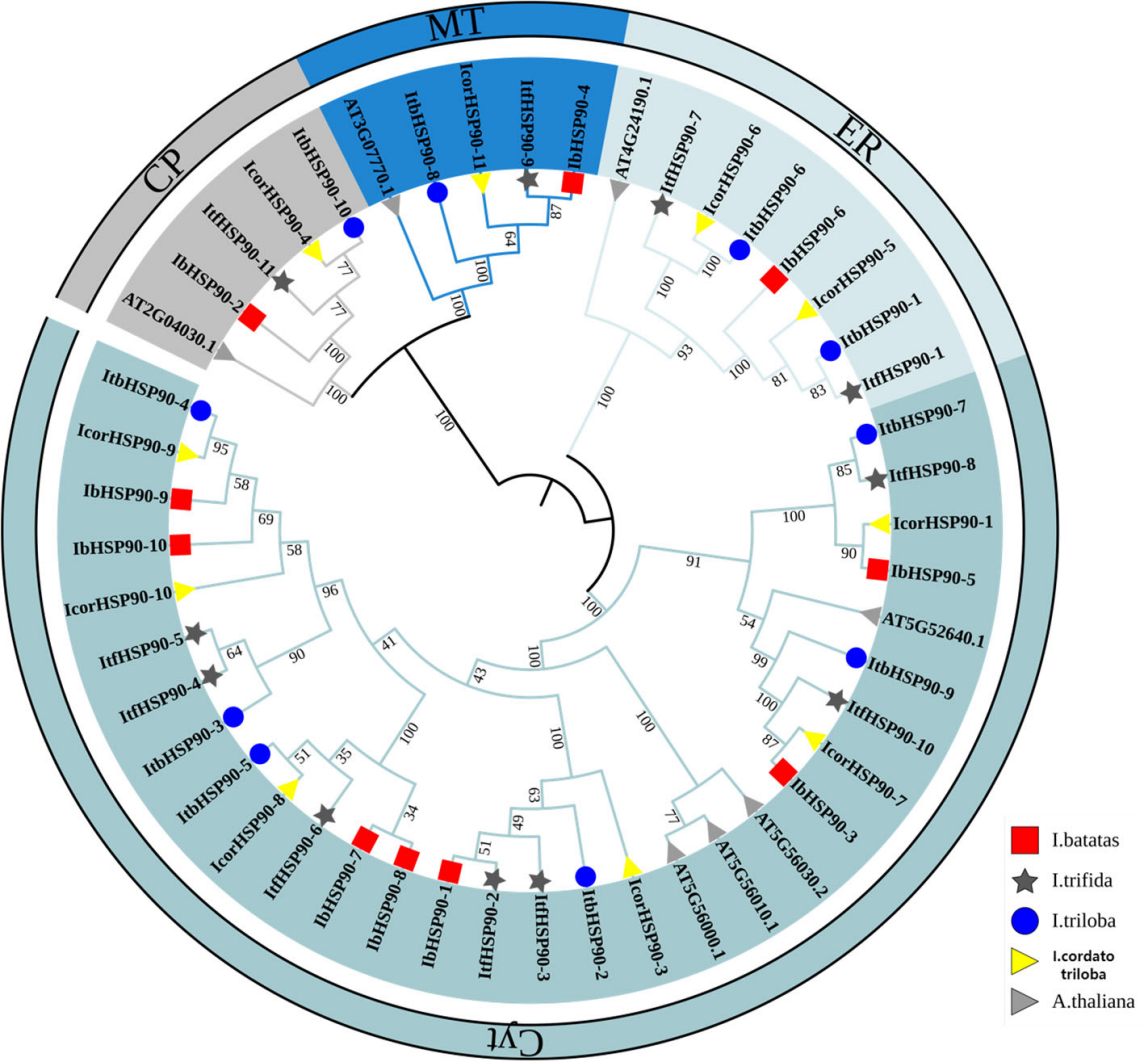
Phylogenetic relationship of HSP90 proteins in *Ipomoea* species and *A. thaliana*. They were classified into four clades according to the bootstrap values.

To elucidate the evolutionary relationships of HSP90s, a rooted maximum-likelihood (ML) phylogenetic tree was constructed using 41 HSP90 protein sequences from four *Ipomoea* species and 7 from *A. thaliana*. HSP90s were categorized into four subfamilies based on topology, aligning with their predicted subcellular localizations: CP, MT, ER and Cyt clades (**Figure 2**). The Cyt clade was the largest, comprising 7 members from *I. batatas*, 7 from *I. trifida*, 6 from *I. triloba*, and 6 from *I. cordatotriloba*. In contrast, the MT and CP clades each contained only a single member per species. These results reveal distinct patterns of copy number variation among the clades, highlighting the diversity of the HSP90 family in *Ipomoea*. Furthermore, the copy number distribution across clades was highly conserved among the four *Ipomoea* species but differed from that in *A. thaliana*, particularly in the ER clade, where *Ipomoea* species maintained more copies. This pattern underscores the evolutionary conservation of *HSP90* genes within the *Ipomoea* genus.

### 3.3 Conserved structural domains and gene structure analysis of *Ipomoea* HSP90s

**Figure 3:**
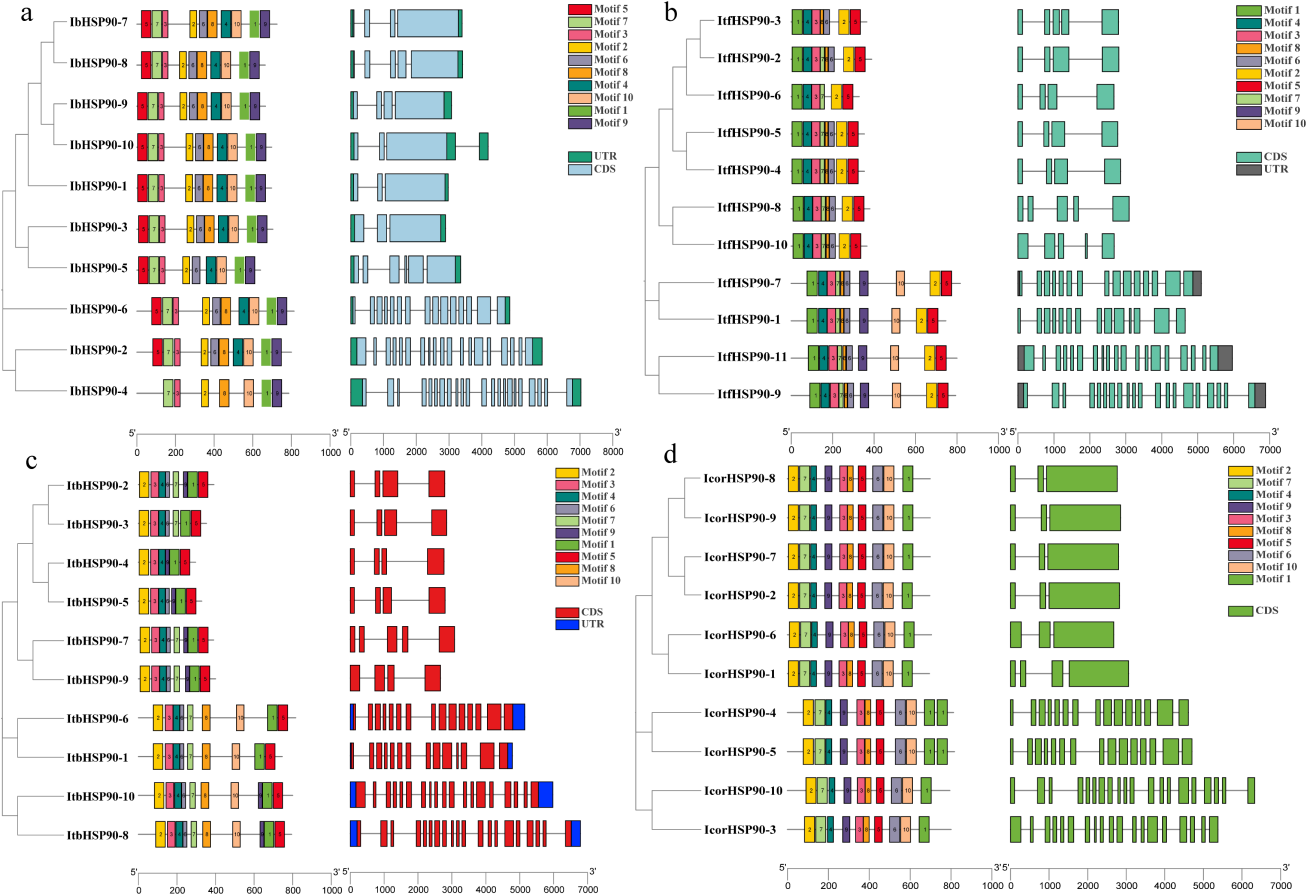
Phylogenetic relationship, gene structure and conserved motif analysis of HSP90s. IbHSP90s **(a)**, ItfHSP90s **(b)**, ItbHSP90s **(c)** and IcorHSP90s **(d)**.

Analysis of conserved motifs revealed variation in composition among the *Ipomoea* HSP90s (**Figure 3**). Most members contained 7 to 10 motifs, while *IcorHSP90–4* and *IcorHSP90–5* possessed 11 motifs. Members within the same phylogenetic clade (e.g., Cyt, ER) generally shared a highly similar motif composition, supporting the subgroup classification and suggesting conserved functional roles. The diversity in motif numbers and arrangements across the family implies a history of sequence divergence following gene duplication events.

Members within the same phylogenetic subgroup consistently shared similar structures, particularly in intron number and exon length. Notably, a distinct set of genes, including *IbHSP90-2, -4, -6*, *ItfHSP90-1, -7, -9, -11*, ItbHSP90-1, -6, -8, -10, and *IcorHSP90-3, -4, -5, -10* were characterized by exceptionally high intron counts, ranging from 13 to 19. In contrast, the majority of other *HSP90* genes possessed a conserved, low-intron architecture, containing only 2 to 5 introns (**Figure 3**). This striking divergence in gene structure, especially the bimodal distribution of intron numbers, provides evidence for structural and potential functional diversification within the *HSP90* gene family across these species.

### 3.4 Evolutionary selection pressure analysis and synteny analysis of *HSP90s* in four *Ipomoea* species

Natural selection pressure serves as a crucial indicator of how plants evolve in response to external stressors. A lower Ka/Ks ratio suggests stronger conservation of amino acid sequences. The analysis of selective pressure on ten IbHSP90s showed Ka/Ks ratios below 1, suggesting purifying selection during their evolution (**Supplementary Table 2**).

**Figure 4:**
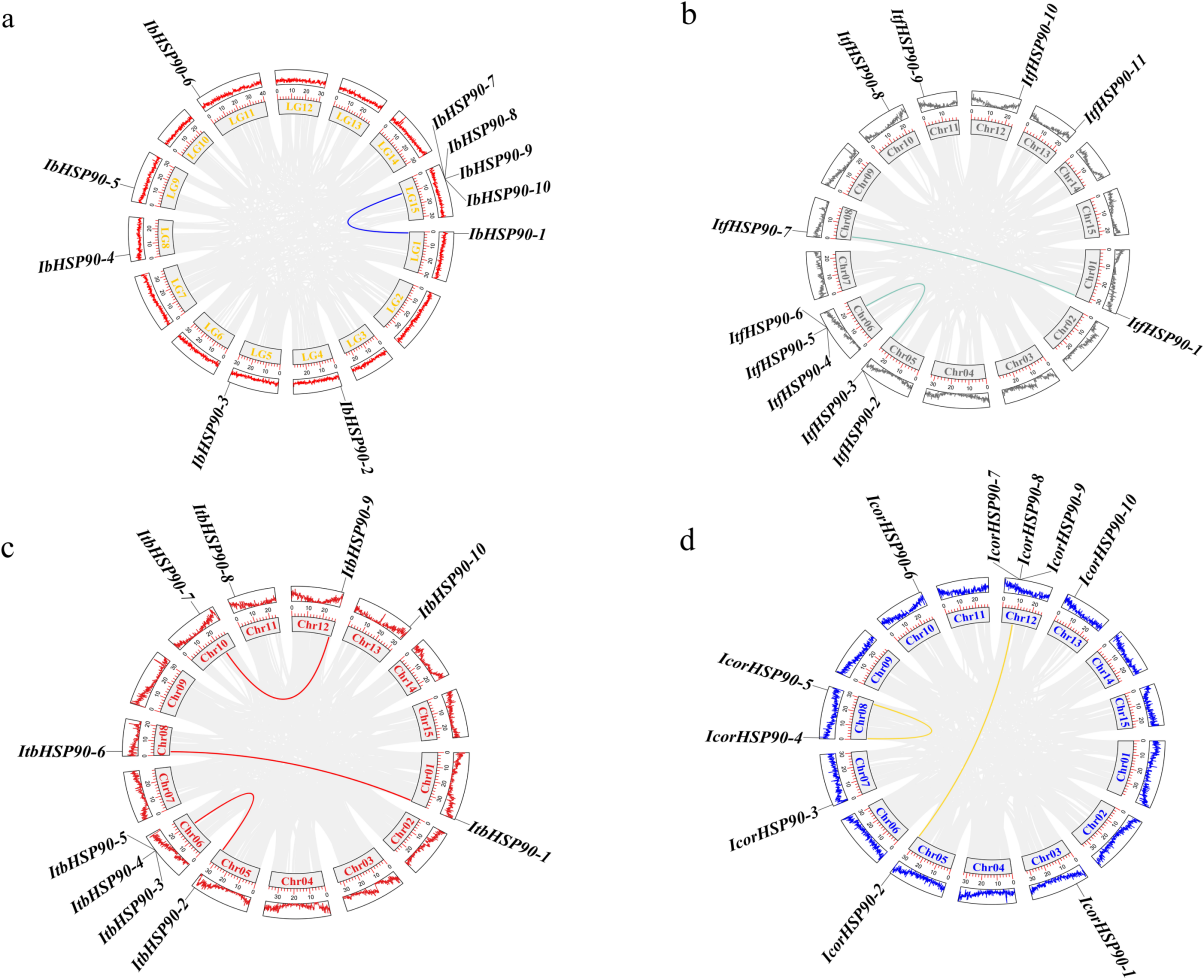
Chromosomal distribution and inter‐chromosomal relationships of *IbHSP90s* **(a)**, *ItfHSP90s* **(b)**, *ItbHSP90s* **(c)** and *IcorHSP90s* **(d)**.

Collinearity analysis identified homologous gene pairs within species as follows:1 in *I. batatas*, 2 in *I. trifida*, 3 in *I. triloba*, and 2 in *I. cordatotriloba* (**Figure 4**). Only *I. cordatotriloba* contained tandemly duplicated *HSP90s* (one pair), the other three species lacked tandem duplications. Segmental duplications were identified as 1 in *I. batatas*, 2 in *I. trifida*, 3 in *I. triloba* and 1 in *I. cordatotriloba* gene pairs. These findings suggest that both segmental and tandem duplications significantly contributed to the expansion of the *HSP90* family in *I. cordatotriloba*, with segmental duplication being the primary factor. In contrast, the other three species depended solely on segmental duplication for *HSP90* amplification.

**Figure 5:**
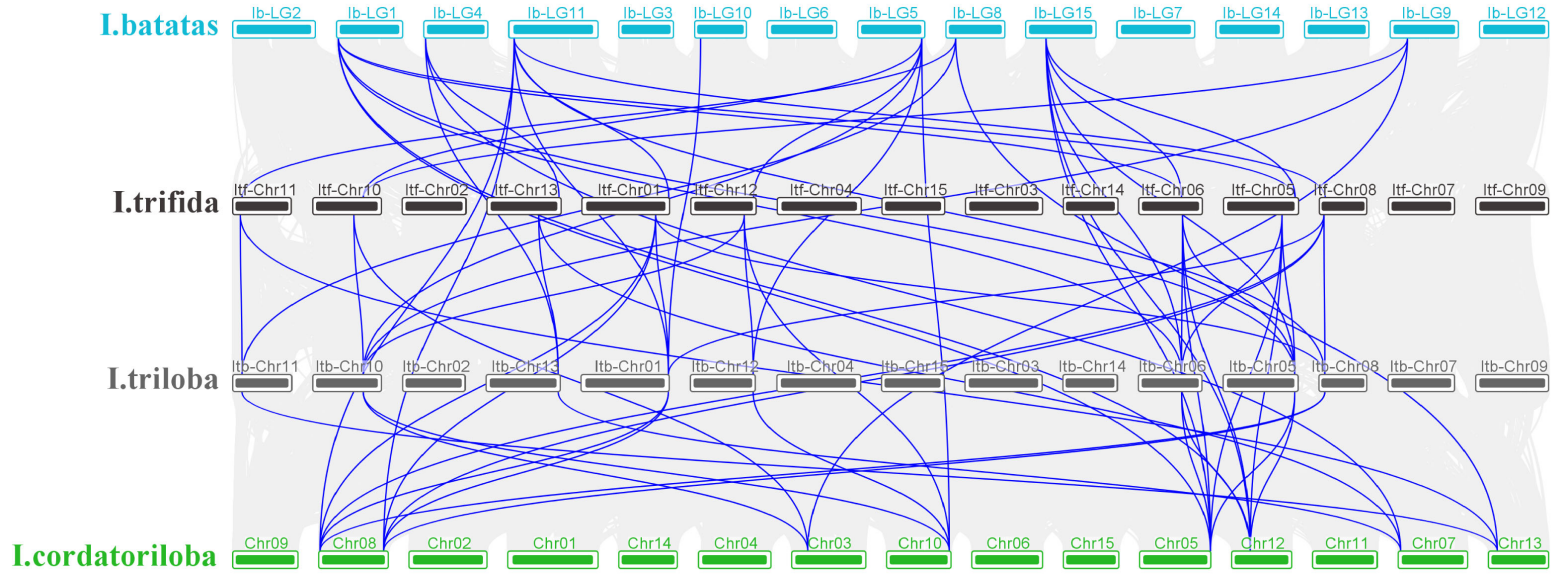
Collinearity analysis of four *Ipomoea* species (*I. batatas, I. trifida, I. triloba, I. cordatotriloba*). Gray lines indicate collinear blocks in the genomes of four *Ipomoea* species. Blue lines highlight syntenic *Ipomoea* species *HSP90* gene pairs.

Interspecies collinearity analysis revealed 74 orthologous *HSP90* pairs across the 41 genes of the four *Ipomoea* species (**Figure 5**). The highest orthologous pair count (13) was observed between *I. triloba*-*I. trifida* and *I. triloba-I.cordatotriloba*, featuring 12 pairs in each of the remaining combinations (*I. batatas*-*I. triloba*, *I. batatas*-*I.cordatotriloba*, *I. trifida*-*I.cordatotriloba*, *I. batatas*-*I. trifida*).

### 3.5 Analysis of cis-acting elements of *IbHSP90s*

**Figure 6:**
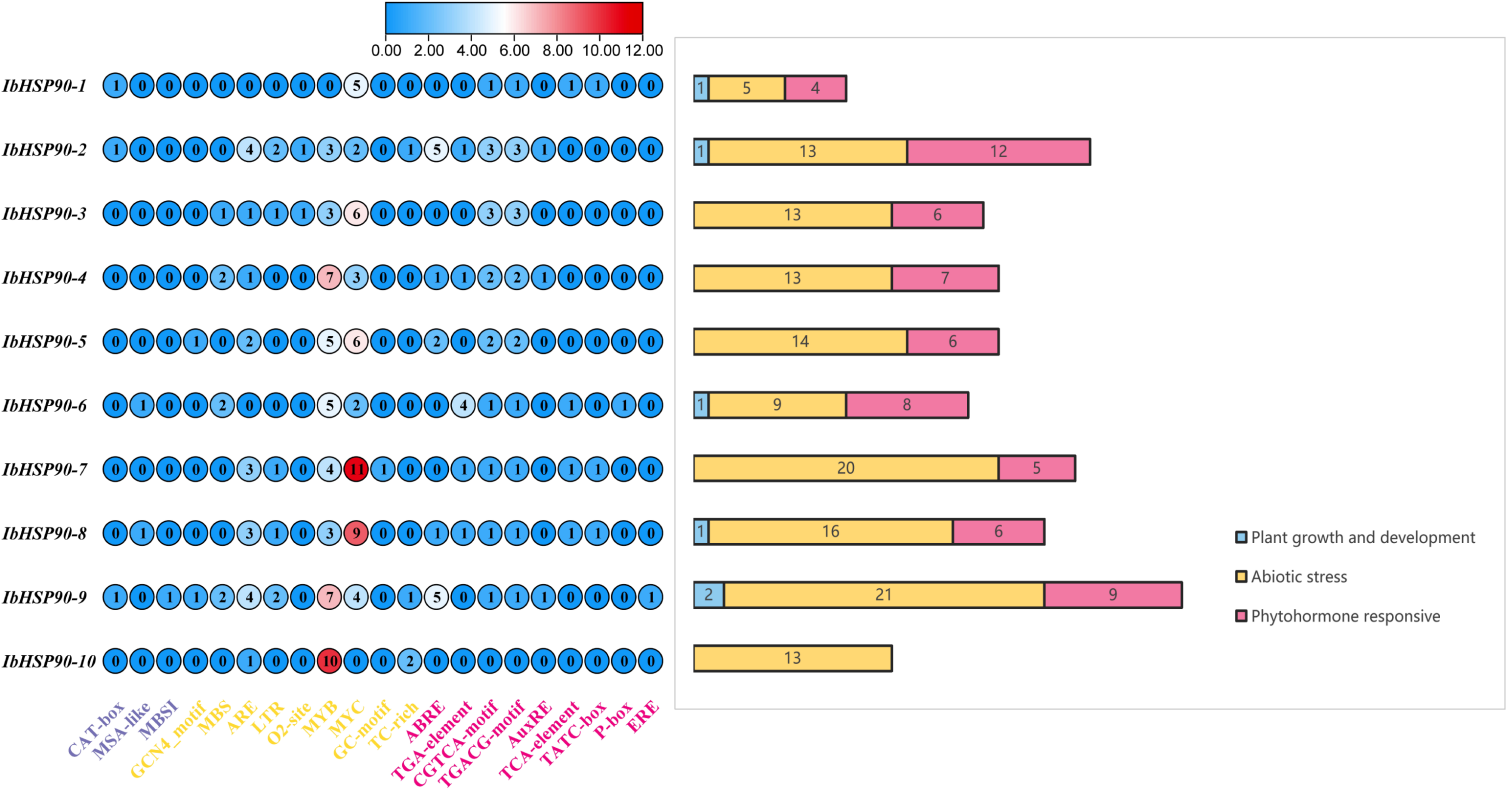
The prediction results of the cis-elements of the *HSP90* gene family in *I. batatas*. The cis-elements analysis was performed with the 2.0 kb upstream region using the online tool PlantCARE.

To clarify the biological processes associated with *IbHSP90s*, we conducted a functional classification of cis-acting elements within their promoter regions. **Figure 6** categorizes these elements into three primary functional groups: plant growth and development, abiotic stress, and phytohormone response. The plant growth and development-related elements include CAT-box, MSA-like (cell cycle-responsive), and MBSI (MYB binding sites regulating flavonoid biosynthesis). CAT-box was the most abundant (3), with MSA-like elements (2) and MBSI (1) following. Abiotic stress-related cis-acting elements included 2 CGN4-motifs, 7 MYB binding sites (MBS), 19 ARE (anaerobic response elements), 7 LTR (low-temperature response elements), 2 O2-sites, 47 MYB elements, 48 MYC elements, 1 GC-motif, and 2 TC-rich elements. Among phytohormone-responsive cis-acting elements: 5 *IbHSP90s* carried ABRE (abscisic acid) and TGA (auxin) response elements, 9 contained MeJA-responsive CGTCA/TGACG-motifs, 3 had AuxRE (auxin) and TATC-box, 4 included TCA (salicylic acid) elements, and only one *IbHSP90* harbored P-box (gibberellin) and ERE (ethylene) elements. These enriched cis-acting elements indicate that *IbHSP90s* may mediate plant responses to diverse stresses and hormonal cues during growth and development, thus regulating adaptation to environmental and internal fluctuations.

### 3.6 Expression analysis of *HSP90s* in sweetpotato and Its two wild diploid relatives

#### 3.6.1 Expression analysis in various tissues

**Figure 7:**
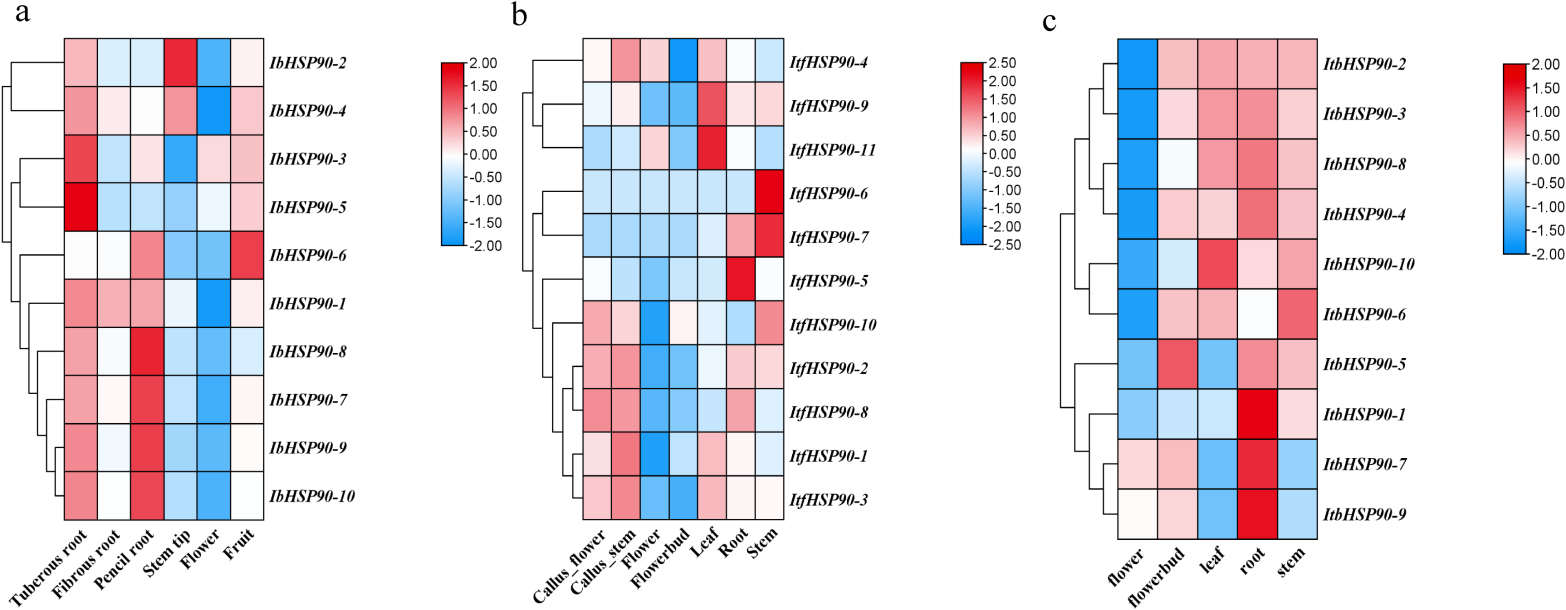
Heatmap of the expression profiles of *HSP90* genes in different tissues of *I. batatas* **(a)**, *I. trifida* **(b)**, *I. triloba* **(c)**. The expression abundance of each transcript is represented by the normalized fragments per kilobase pair per million (FPKM) value and displayed as colored boxes from green (lower expression) to red (higher expression).

**Figure 8:**
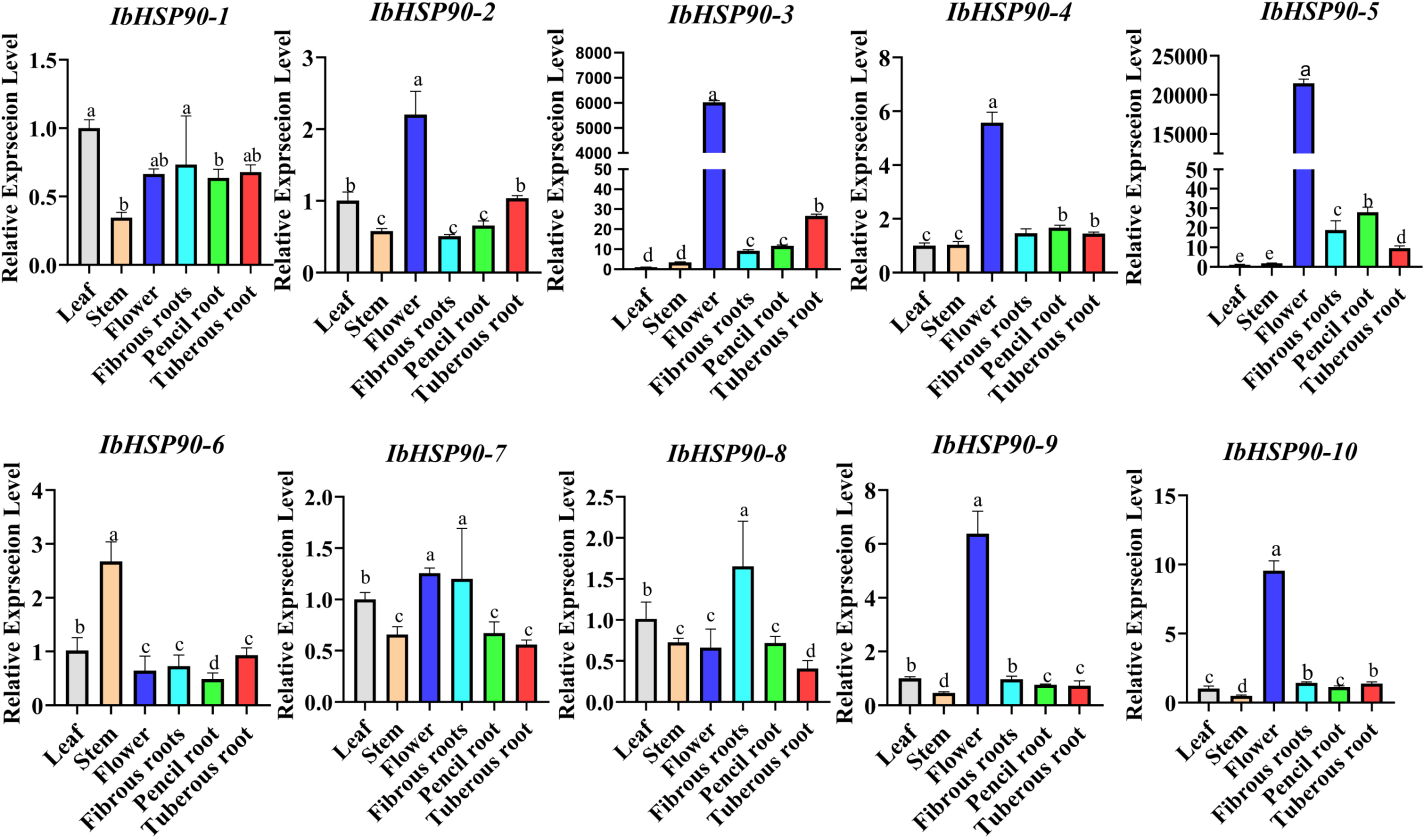
Relative expression levels of 10 *IbHSP90s* in 6 different tissues of the sweetpotato. The x-axes represent different tissues, including leaf, stem, flower, fibrous root, pencil root, tuberous root; the y-axes indicate the relative expression of *IbHSP90s*; the error bars depicted represent the standard errors calculated from three technical replicates obtained from a single bulked biological sample.

To investigate the expression profiles of *IbHSP90s*, transcriptomic data derived from six distinct organs (tuberous root, fibrous root, pencil root, stem tip, flower, fruit) of Jishu26 were analyzed. All *IbHSP90s*, except *IbHSP90-6*, showed relatively high expression levels in the tuberous roots. Over 50% of *IbHSP90s* showed elevated expression levels in fibrous roots. *IbHSP90–2* and *IbHSP90–6* exhibited high expression levels in the stem tip and fruit, respectively (**Figure 7a**). To further validate the expression pattern of *IbHSP90s* in various tissues of sweetpotato, we conducted qRT-PCR. The study found that IbHSP90s expression levels differ among tissues, with *IbHSP90-2*, *IbHSP90-3*, *IbHSP90-4*, *IbHSP90-5*, *IbHSP90-9*, and *IbHSP90–10* showing high expression in sweetpotato flowers. *IbHSP90–6* and *IbHSP90–7* showed strong upregulated expression in the stem and fibrous root, respectively. *IbHSP90–1* and *IbHSP90–8* exhibited similar expression patterns and were down-regulated in the stem (**Figure 8**). Transcriptome analysis of seven tissues (Callus_flower, Callus_stem, flower, flowerbud, leaf, stem, root) in *I. trifida* and five tissues (flower, flowerbud, leaf, stem, root) in *I. triloba* revealed that approximately half of the *ItfHSP90s* members, including *ItfHSP90-10*, *ItfHSP90-2*, *ItfHSP90-8*, *ItfHSP90-1*, *ItfHSP90-3*, and *ItfHSP90–4* showed increased expression in callus_flower and callus_stem in *I. trifida*. Moreover, *ItfHSP90–9* and *ItfHSP90–11* showed significantly increased expression in leaf tissue, while *ItfHSP90–6* and *ItfHSP90–7* were highly expressed in stem (**Figure 7b**). Notably, *ItfHSP90–5* was exclusively and significantly expressed in root tissue. For the *ItbHSP90s*, the majority of members were highly expressed in root and stem tissues. *ItbHSP90–5* and *ItbHSP90–10* showed peak expression levels in flowerbud and leaf, respectively (**Figure 7c**). The findings indicate that although HSP90s show unique expression patterns in various tissues, their homologous genes have evolved distinct functions in sweetpotato and its two diploid relatives.

#### 3.6.2 Analysis of hormonal response expression

**Figure 9:**
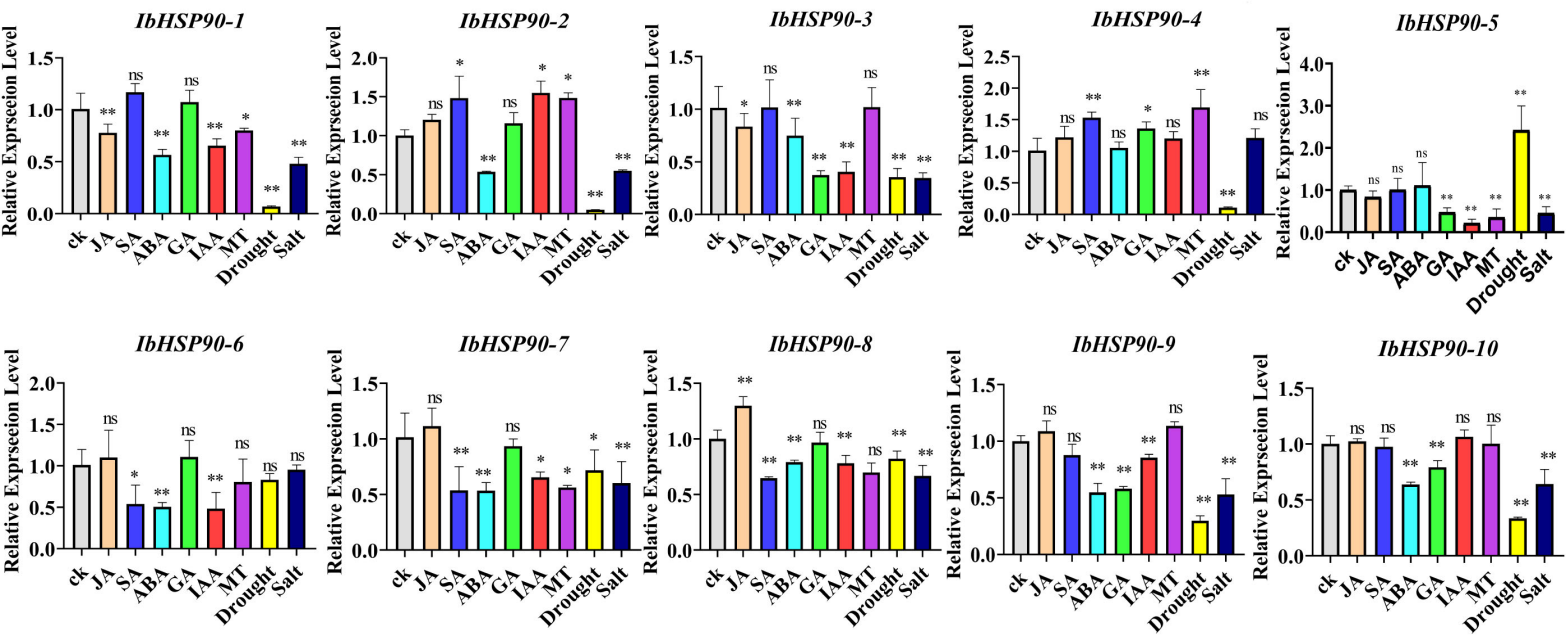
Analysis of qRT-PCR of *IbHSP90* family genes in sweetpotato under hormonal treatment (JA, SA, ABA, GA, IAA, MT),drought and salt stress. Error bars indicate the standard error of the mean (n = 3). The significance analysis was performed by one-way ANOVA method, the significance mark (*) indicating P < 0.05 and (**) indicating P < 0.001.

The expression profiles of *IbHSP90s* under phytohormone treatments (JA, SA, ABA, IAA, GA, MT) were quantitatively assessed by qRT-PCR, indicating that expression of most *IbHSP90s* was suppressed by hormonal treatments, with the exception of *IbHSP90–2* and *IbHSP90-4*, which were induced (**Figure 9**). Transcriptomic analysis revealed that approximately half of the *ItfHSP90s* in *I. trifida* were upregulated by 6-benzylaminopurine and GA3 treatment. Conversely, IAA application led to broad suppression of *ItfHSP90s* expression, with the exception of *ItfHSP90-9*. Furthermore, the expression of *ItfHSP90–1* and *ItfHSP90–10* was coordinately induced by ABA treatment (**Supplementary Figure 2a**). In *I. triloba*, the majority of *ItbHSP90s* were activated by ABA and GA3, except for *ItbHSP90-1*, which was inhibited by all four hormone treatments (**Supplementary Figure 2b**). The findings indicate that homologous genes in sweetpotato and its two diploids exhibit varied responses to hormone treatments, suggesting that *IbHSP90s*, *ItfHSP90s* and *ItbHSP90s* may participate in distinct hormonal pathways.

#### 3.6.3 Expression analysis under abiotic stresses

**Figure 10:**
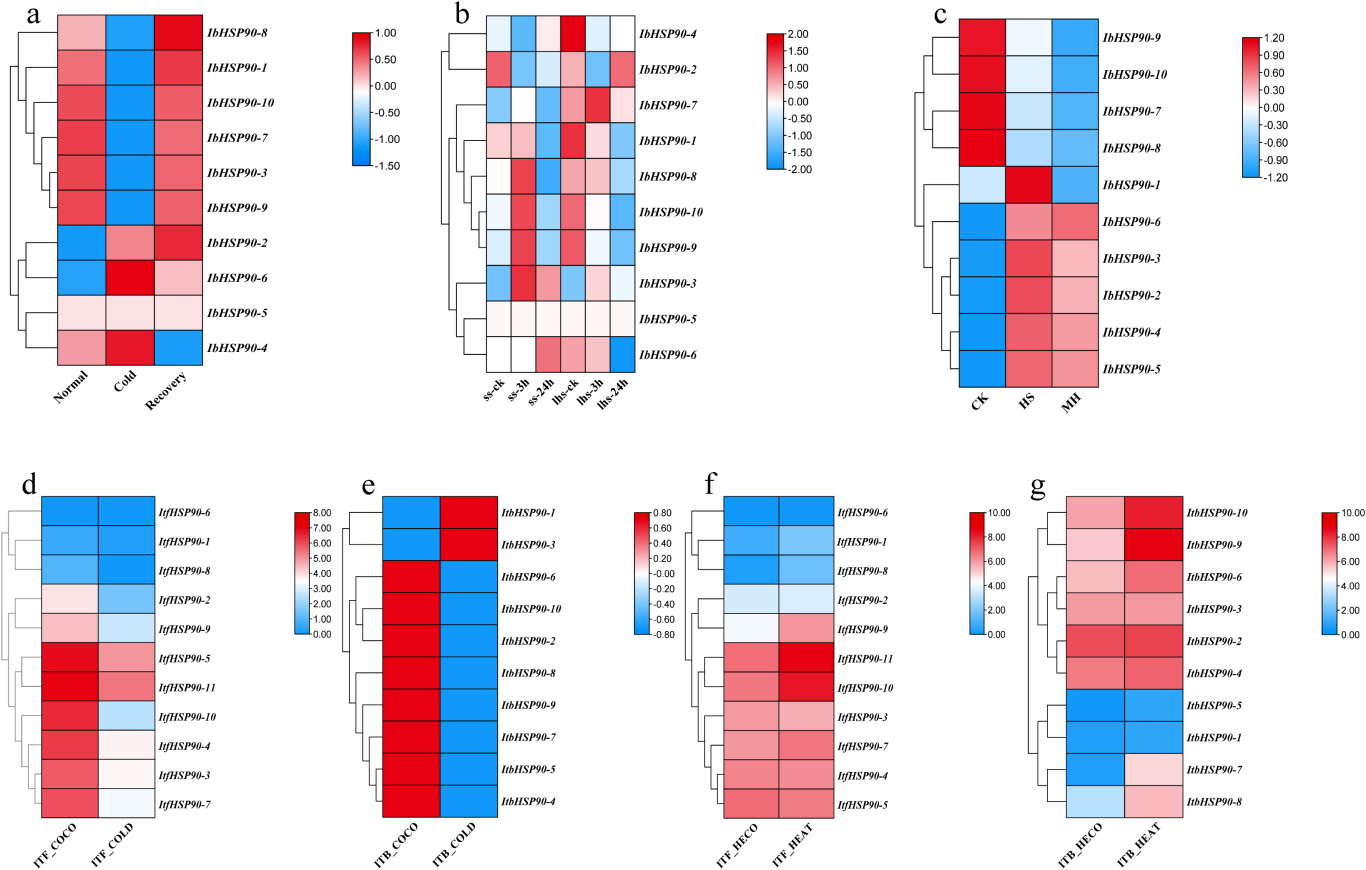
Expression analysis of *HSP90s* in *I. batatas*, *I. trifida* and *I. triloba* under cold and heat stresses as determined by RNA-seq. **(a)** Expression analysis of *IbHSP90s* in the Sinhwangmi under cold stress. **(b)** Expression analysis of *IbHSP90s* in the Shenshu 28(ss) and Liaohanshu 21(lhs) under cold stress. **(c)** Expression analysis of *IbHSP90s* in *I. batatas* under heat stress. HS, heat stress; MH, Heat treatment after melatonin application. **(d)** Expression analysis of *ItfHSP90s* in *I. trifida* under cold stress. ITF_COCO: *Ipomoea trifida* cold control 28/22-deg C day/night experiment. ITF_COLD, Ipomoea trifida cold stress 10/4-deg C day/night experiment; **(e)** Expression analysis of *ItbHSP90s* in *I. triloba* under cold stress. ITB_COCO, *Ipomoea triloba* cold control 28/22-deg C day/night experiment; ITB_COLD, *Ipomoea triloba* cold stress 10/4-deg C day/night experiment; **(f)** Expression analysis of *ItfHSP90s* in *I. trifida* under heat stress. ITF_HECO, *Ipomoea trifida* heat control 28/22-deg C day/night experiment; ITF_HEAT, *Ipomoea trifida* heat stress 35/35-deg C day/night experiment; **(g)** Expression analysis of *ItbHSP90s* in *I. triloba* under heat stress. ITB_HECO, *Ipomoea triloba* heat control 28/22-deg C day/night experiment; ITB_HEAT, *Ipomoea triloba* heat stress 35/35-deg C day/night experiment.

**Figure 11:**
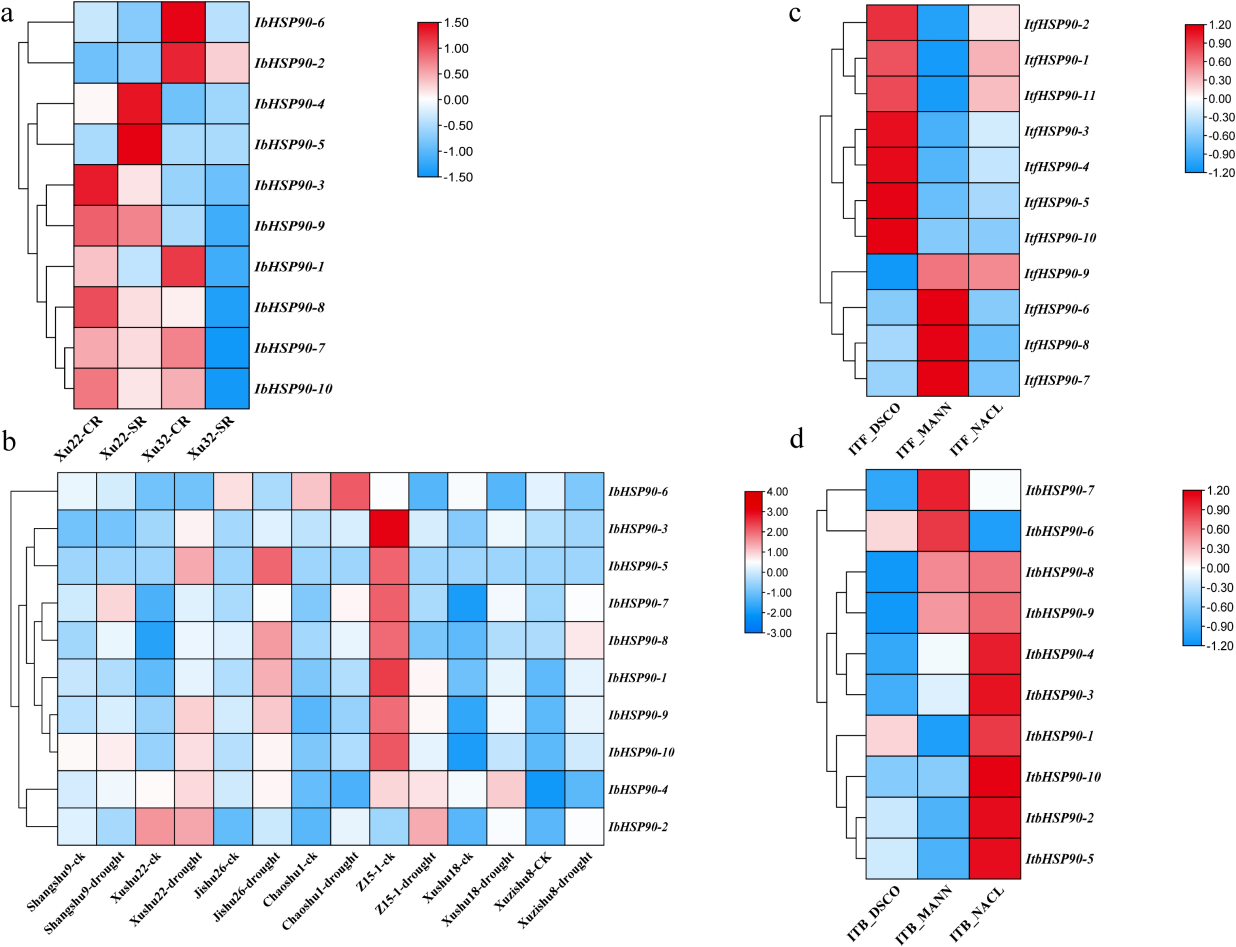
Expression analysis of *HSP90s* in *I. batatas*, *I. trifida* and *I. triloba* under salt and drought stresses as determined by RNA-seq. **(a)** Expression analysis of *IbHSP90s* in the Xushu 22 and Xushu 32 under salt stress. **(b)** Expression analysis of *IbHSP90s* under drought stress. **(c)** Expression analysis of *ItfHSP90s* in *I. trifida* under salt and drought stress. ITF_DSCO, *Ipomoea trifida* drought and salt control experiment; ITF_MANN, *Ipomoea trifida* mannitol drought stress experiment; ITF_NACL, *Ipomoea trifida* NaCl salt stress experiment; **(d)** Expression analysis of *ItbHSP90s* in *I. triloba* under salt and drought stress. ITB_DSCO, *Ipomoea triloba* drought and salt control experiment; ITB_MANN, *Ipomoea triloba* mannitol drought stress experiment; ITB_NACL,*Ipomoea triloba* NaCl salt stress experiment.

**Figure 12:**
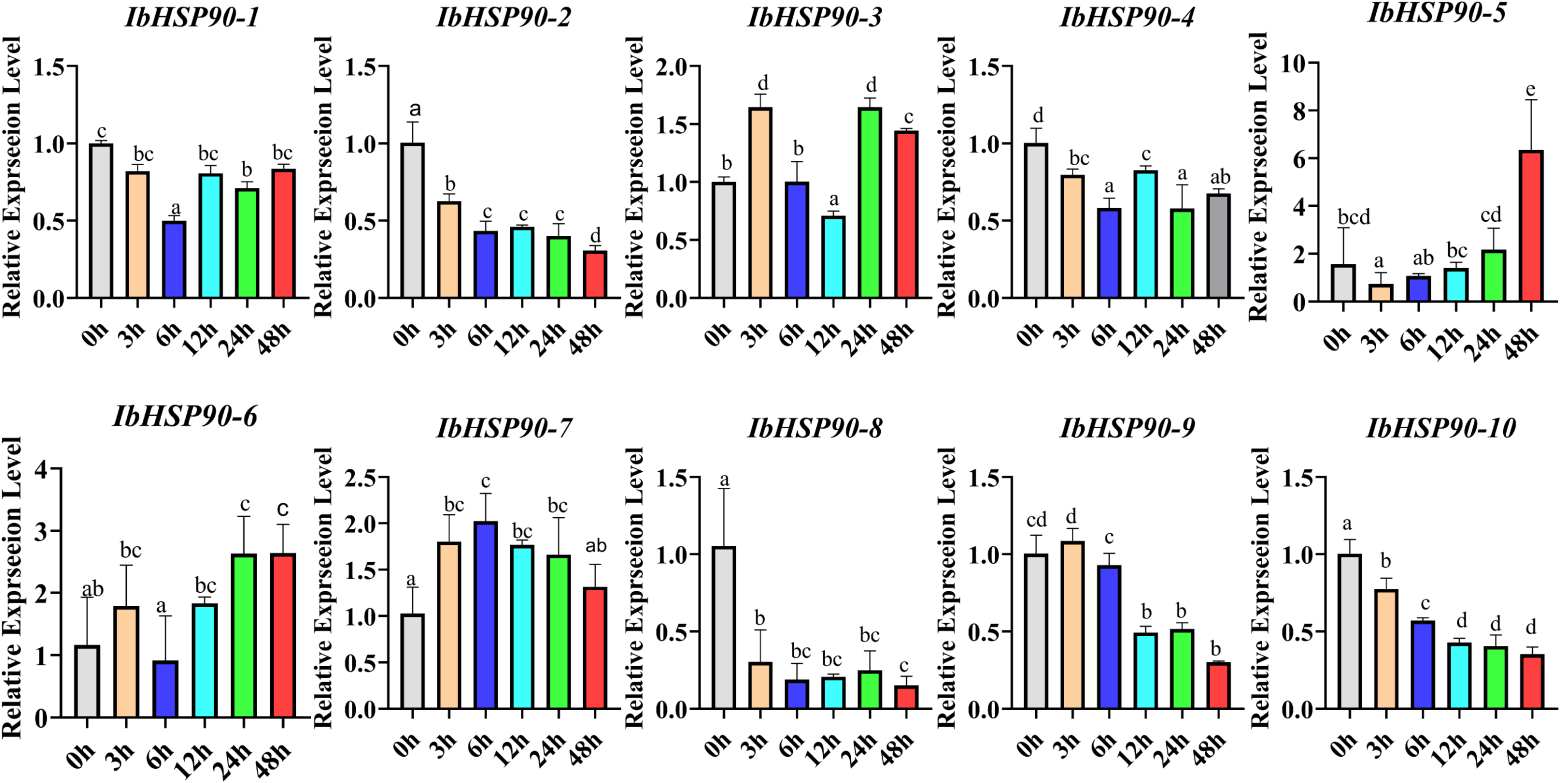
Changes in the expression levels of 10 *IbHSP90* genes under cold treatment. The different letters of a, b, c, and d indicate significant differences at p < 0.05, as determined by one-way ANOVA with SPSS single-factor tests.

The study analyzed transcriptome data to assess *IbHSP90s* expression patterns under various abiotic stress conditions: cold treatment (4 °C) in Sinhwangmi, cold stress (4 °C) in the cold-sensitive cultivar Shenshu 28 and the cold-tolerant line Liaohanshu 21, heat stress in Haida 7791, NaCl treatment (200 mM) in the salt-sensitive cultivar Xushu 32 and the salt-tolerant line Xushu 22, and PEG (30%) treatment in seven sweetpotato cultivars with differing drought resistance. In Sinhwangmi, the expression of most *IbHSP90s* was down-regulated under cold stress but restored to normal levels after recovery at 25°C (**Figure 10a**). At normal temperatures, Liaohanshu 21 exhibited significantly higher *IbHSP90* expression levels compared to Shenshu 28. However, after 3 hours of low-temperature treatment, the expression of *IbHSP90s* in Shenshu 28 increased markedly, whereas it was suppressed in Liaohanshu 21 (**Figure 10b**). Upon sustained low-temperature exposure, the majority of *IbHSP90s* in both cultivars were down-regulated. However, heat treatment induced the expression of *HSP90s*, but this up-regulation was partially suppressed by melatonin treatment (**Figure 10c**). Without NaCl treatment, more than half of the *HSP90s* in the salt-tolerant variety Xushu 22 exhibited higher expression levels than those in the salt-sensitive variety Xushu 32. However, *IbHSP90–6* and *IbHSP90–2* showed an opposite expression trend. In addition, most *HSP90s* in both Xushu 22 and Xushu 32 were down-regulated after NaCl treatment. In contrast, *IbHSP90–4* and *IbHSP90–5* in Xushu 22 were markedly induced by NaCl stress (**Figure 11a**). Under PEG treatment, *IbHSP90s* expression remained stable, but was inhibited in the drought-tolerant cultivar Z15-1 (**Figure 11b**), indicating cultivar-specific variations in *IbHSP90s* response to drought stress. We conducted qRT-PCR analysis to confirm the expression patterns of IbHSP90s under cold, NaCl, and PEG treatments. The results revealed that most *IbHSP90s* were down-regulated following cold treatment (**Figure 12**). Both NaCl and PEG treatments led to a similar suppression trend in the expression of most IbHSP90s (**Figure 9**).

RNA-seq analysis was conducted to determine the expression patterns of *ItfHSP90s* and *ItbHSP90s* in *I. trifida* and *I. triloba* under cold, heat, PEG and NaCl treatments. While the majority of *ItfHSP90s* were down-regulated by cold treatment, they were up-regulated under heat stress (**Figures 10d, f**). Under both NaCl and PEG treatments, the majority of ItfHSP90s were down-regulated. In contrast, *ItfHSP90–9* was up-regulated under both stress conditions. Additionally, *ItfHSP90-6*, *ItfHSP90–7* and *ItfHSP90–8* were specifically induced only under drought (PEG) stress (**Figure 11c**). *ItbHSP90* members exhibited expression patterns similar to those of *ItfHSP90s* under cold, and heat treatments (**Figures 10e, g**). Notably, a differential response was observed under NaCl stress, with the majority of *ItbHSP90s* being induced (**Figure 11d**).

### 3.7 Protein-protein interaction network and KEGG functional enrichment analysis of IbHSP90s

The protein-protein interaction (PPI) network of the HSP90s was constructed using the STRING database. Notably, IbHSP90 isoforms exhibit dense interactions with two classic chaperone co-factors:HSP Organizing Protein (HOP), represented by HOP2 and HOP3, which act as key mediators facilitating client protein recruitment to HSP90 complexes. Suppressor of G2 allele of Skp1 (SGT1), including SGT1A and SGT1B, which function as co-chaperones implicated in protein folding and immune signaling cascades. Additionally, IbHSP90s interact with the heat shock transcription factor HSFA2, a critical regulator of stress-responsive gene expression. This interaction pattern suggests that IbHSP90s may coordinate with HOP, SGT1, and HSFA2 to execute their central chaperone functions and regulate stress responses in sweetpotato (**Supplementary Figure 3**). Collectively, these observations indicate that the IbHSP90-centered interaction network modulates plant growth, development, and stress adaptation.

We conducted KEGG pathway enrichment analysis to elucidate the functional pathways and potential roles of IbHSP90s. Results revealed significant enrichment in endoplasmic reticulum processing pathways, chaperone and folding catalyst functions, indicating their potential role in protein folding, quality control, and stress responses to maintain cellular homeostasis (**Supplementary Figure 4**). Furthermore, the HSP90 family was significantly enriched in environmental adaptation pathways, pointing to its potential involvement in plant abiotic stress responses.

### 3.8 Subcellular localization of IbHSP90-2

**Figure 13:**
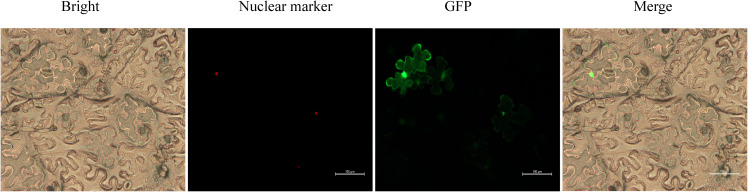
Subcellular localization of IbHSP0–2 in *N. benthamiana* using red fluorescent protein as markers for nuclear localization, of IbHSP90–2 GFP represents green fluorescent protein, Bright represents visible light, and Merge represents the combination of GFP and the marker.

Subcellular localization is pivotal for protein function, as it dictates cellular roles. Prolonged low-temperature treatment suppressed IbHSP90–2 expression, implying its potential involvement in sweetpotato cold responses. To clarify its function, we analyzed IbHSP90–2 subcellular localization by constructing the p1300-NLS-Red-GFP-IbHSP90–2 vector. The construct was introduced into *Agrobacterium* and transiently expressed in *N.benthamiana* leaves through co-infiltration. GFP fluorescence localized to both the nucleus and cytoplasm, while the nuclear marker NLS-DsRed was restricted to the nucleus (**Figure 13**). These findings confirm IbHSP90-2’s dual localization, indicating potential functional roles across multiple cellular compartments.

### 3.9 Cold stress expression of IbHSP90–2 in yeast

**Figure 14:**
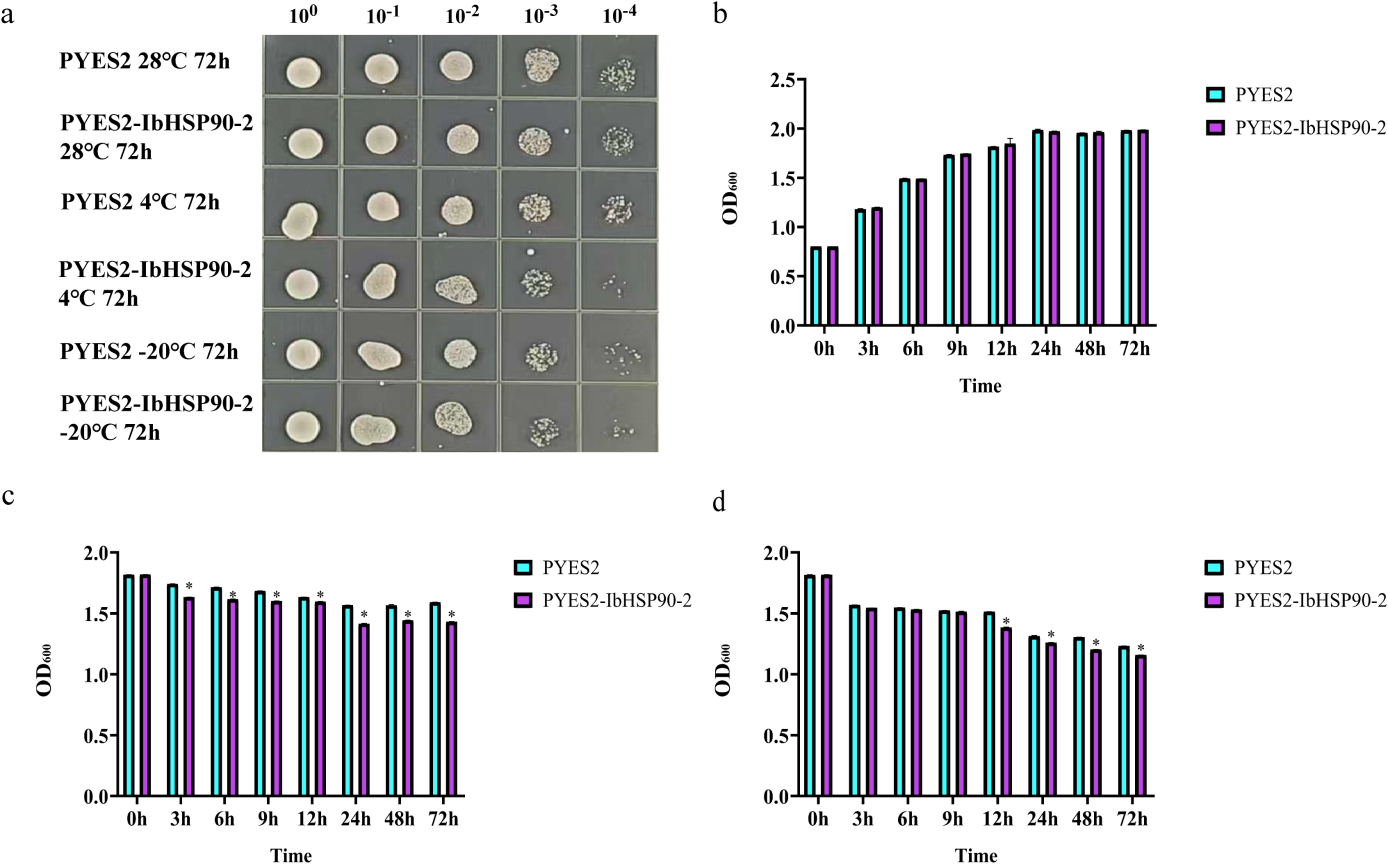
The function analysis of IbHSP90–2 under cold stress in yeast strain INVSc1. **(a)** Serially diluted drops of yeast transformants with or without cold treatment. **(b)** OD_600_ values of yeast at 28°C. **(c)** OD_600_ values of yeast at 4 °C. **(d)** OD_600_ values of yeast at -20°C.The (asterisk) symbol indicates a significant difference compared with the control at P < 0.05 (F test).

To investigate the role of IbHSP90–2 in cold stress responses, control (INVSc1-pYES2) and recombinant (INVSc1-pYES2-IbHSP90-2) yeast strains were induced, cultured to the same cell densities (OD600 = 0.8) and subsequently exposed to 28°C, −4°C, and −20°C. Under non-stress conditions (28°C), growth of the recombinant strain was similar to that of the control (**Figure 14a**), indicating that heterologous expression of *IbHSP90–2* had no significant effect on yeast growth. Under low-temperature stress, however, the control strain grew markedly better than the IbHSP90–2 overexpressing strain, and the difference became more pronounced with increasing dilution ratios (**Figure 14a**). To quantify growth, cell density (OD600) was monitored over time. Under non-stress conditions, OD600 values of the control and recombinant strains were not significantly different (P > 0.05, **Figure 14b**). In contrast, under low-temperature stress, OD600 values of the control strain were significantly higher than those of the recombinant strain (P < 0.05, **Figures 14c, d**). These results demonstrate that overexpression of IbHSP90–2 significantly reduces low-temperature tolerance in yeast cells.

## 4 Discussion

While the *HSP90* gene family has been extensively studied across a wide range of plant species, and accumulating evidence highlights its essential roles in mediating responses to various abiotic and biotic stresses including extreme temperatures, salinity, drought, and pathogen infections, a systematic and comprehensive investigation of this gene family in sweetpotato remains notably absent (Milioni and Hatzopoulos, 1997; Zhang et al., 2013; Song et al., 2019; Bettaieb et al., 2020; Li et al., 2020; Wei et al., 2020; Peng et al., 2024; Wei et al., 2024; Xu et al., 2024; Hao et al., 2025; Xiao et al., 2025). Sweetpotato is an economically important crop globally, valued primarily for its nutrient-rich storage roots. It diverged from its diploid wild relatives, *I. trifida* and *I. triloba*, and both species possess a chromosome complement of 2n = 2x = 30 (Hu et al., 2024; Peng et al., 2025). Furthermore, *I. cordatotriloba* is a rare exception among sweetpotato wild relatives because it also develops storage roots, which makes it a valuable model for investigating the genetic basis of storage root formation (Xiao et al., 2025). To fill this research gap and gain insights into the evolutionary dynamics and functional divergence of *HSP90* genes in sweetpotato and its relatives, we conducted a genome-wide identification of the *HSP90* gene family across these four *Ipomoea* species. Our analysis identified a total of 41 *HSP90* genes: 10 in *I. batatas*, 11 in *I. trifida*, 10 in *I. triloba*, and 10 in *I. cordatotriloba*. Notably, the *HSP90* gene family is highly conserved among these four species. Three of them (*I. batatas*, *I. triloba*, and *I. cordatotriloba*) harbor 10 *HSP90* genes each, while *I. trifida* contains an additional copy with 11 genes in total. This difference may reflect a recent gene duplication event specific to *I. trifida* during its evolutionary divergence. Although *I. trifida* and *I. triloba* exhibit a conserved chromosomal distribution pattern of *HSP90* genes, significant differences were observed across all four *Ipomoea* species (**Figure 1**), and these differences may be associated with species-specific chromosomal rearrangements and functional specialization during evolution. Analysis of the physicochemical properties of HSP90 proteins revealed diverse amino acid lengths, molecular weights, and pI values among the four species (**Supplementary Figure 1**). Consistent with the conserved chaperone function of HSP90 proteins, most IbHSP90s and their homologs were predicted to be stable and hydrophobic. They are primarily localized in the endoplasmic reticulum or cytoplasm (**Supplementary Table 1**), and these subcellular compartments align with their well-documented roles in protein folding, intracellular signaling, and stress response mediation.

Based on phylogenetic analysis with *A.thaliana* HSP90 proteins, the identified HSP90s from the four *Ipomoea* species were categorized into four distinct classes (**Figure 2**), and this result reflects evolutionary conservation between sweetpotato and the model plant *A.thaliana*. Evolutionary analysis further revealed divergent mechanisms driving HSP90 family expansion. In *I. cordatotriloba*, HSP90 amplification was mediated by both segmental and tandem duplications, and segmental duplication acts as the predominant mechanism. In contrast, HSP90 expansion in *I. batatas*, *I. trifida*, and *I. triloba* occurred exclusively through segmental duplication (**Figures 4**, **5**). This distinction suggests that *I. cordatotriloba* may have evolved unique adaptive strategies involving HSP90 genes. Numerous studies have demonstrated a close correlation between plant responses to phytohormones and environmental stresses (Ciura and Kruk, 2018, Bittner et al., 2022; Guo et al., 2024). To explore the potential regulatory roles of *IbHSP90* genes in these processes, we screened the promoters of all *IbHSP90* genes for cis-regulatory elements (CREs) and identified a total of 204 CREs. Among these, only 6 were associated with development, while 198 were related to stress responses or phytohormone signaling (**Figure 6**). These elements account for approximately 97% of all identified CREs. This striking enrichment of CREs related to stress and hormones strongly suggests that *IbHSP90* genes play pivotal roles in sweetpotato’s growth and development, and more notably, are involved in adaptation to adverse environmental conditions. Taken together, these findings lay a comprehensive foundation for subsequent functional characterization of the *HSP90* gene family in sweetpotato and related species, particularly with respect to stress tolerance.

*HSP90s* exhibits differential expression across various plant tissues, with abundant levels in flowers and leaves but significantly lower levels in root and stem tissues (Li et al., 2020). However, *HSP90* genes in *Ipomoea* species generally show high expression levels in floral, root, and stem tissues, which indicates divergent expression patterns of HSP90 family members among different plant taxa (**Figures 7a**, **10**). Phytohormones serve as key regulators in plant stress responses (Jogawat et al., 2021; Gogoi et al., 2024). Previous studies have documented that HSP90 genes participate in auxin signaling and are transcriptionally induced by ABA treatment (Song et al., 2019; Samakovli et al., 2021). Conversely, auxin treatment led to the down-regulation of all *ItfHSP90* genes except *ItfHSP90-9* (**Supplementary Figure 2a**), whereas the majority of *ItbHSP90* genes were up-regulated in response to ABA (**Supplementary Figure 2b**). Notably, a stronger inhibitory effect was detected in sweetpotato, as multiple *IbHSP90* genes were repressed by various phytohormone treatments (**Figure 12**). Collectively, these observations suggest that *HSP90* genes may mediate species-specific regulatory modules within phytohormone signaling pathways. The *HSP* gene family is deeply involved in plant thermotolerance (Hahn et al., 2011; Huang et al., 2019). Consistent with this, most *HSP90* genes in *Ipomoea* species display induced expression under high-temperature stress (**Figures 10c, f, g**). Although functional studies of HSP90 in low-temperature responses are still relatively limited, increasing evidence has indicated its involvement in cold adaptation in several plant species. For instance, three *HSP90* genes contribute to cold stress responses in alfalfa, and six *HSP90* genes participate in cold stress regulation in cabbage (Sajad et al., 2022; Liu et al., 2025). Moreover, differential expression of HSP90 genes in ginger and barley under cold conditions also supports a potential regulatory role in this process (Sadura et al., 2020; Stachurska et al., 2024; Xiao et al., 2025). In sweetpotato, however, expression profiling under cold stress revealed broad down-regulation of HSP90 transcripts (**Figures 10a, b**, **12**), suggesting that *IbHSP90* genes may act as negative regulators in cold-response signaling pathways. HSP90 proteins are also critical for plant adaptation to drought and salt stresses. In cassava, the molecular chaperone MeHSP90 promotes drought resistance by facilitating the assembly of MeWRKY20 and MeCatalase1 complexes (Wei et al., 2020). Meanwhile, salt stress can induce HSP90 transcription, but overexpression of *AtHsp90.2*, *AtHsp90.5*, and *AtHsp90.7* in *A.thaliana* enhances plant sensitivity to both salt and drought stresses (Song et al., 2009). Altogether, *HSP90* genes exhibit species-specific expression patterns in response to drought and salt stresses across *Ipomoea* species. Specifically, most HSP90 members in sweetpotato and *I. trifida* were down-regulated under both PEG and NaCl treatments, whereas *ItbHSP90* genes were induced by NaCl stress (**Figures 8**, **11c, d**).

Accumulating studies have demonstrated that HSP90 proteins exert their regulatory functions primarily through interactions with a suite of partner proteins, including heat shock factor (HSF) transcription factors, as well as the co-chaperones Hsp70-Hsp90 organizing protein (HOP) and suppressor of G2 allele of SKP1 (SGT1) (Botër et al., 2007; Hahn et al., 2011; Castellano et al., 2024). HOP serves as a critical adaptor that facilitates the conformational maturation of HSP90, while SGT1 interacts with HSP90 to modulate protein complex assembly and signal transduction, particularly in plant stress responses (Yuan et al., 2021). In the present study, we predicted protein-protein interactions (PPIs) between HSP90 and these partner proteins using the STRING database to explore the potential regulatory network of IbHSP90. Consistent with previous findings, our results revealed strong interactions between IbHSP90 proteins and members of the HSF-A2 subfamily (**Supplementary Figure 3**). Additionally, we detected significant interactions between IbHSP90 and both HOP and SGT1 proteins. These observations suggest that IbHSP90 may form functional complexes with HOP, SGT1, and specific HSF-A2 members to synergistically regulate downstream stress-responsive signaling pathways in sweetpotato, providing a preliminary framework for understanding the molecular mechanisms underlying IbHSP90-mediated environmental adaptation. To further explore the potential biological functions of *IbHSP90* genes, we performed KEGG pathway enrichment analysis. The results showed that *IbHSP90* genes were significantly enriched in the endoplasmic reticulum protein processing pathway, which is consistent with their conserved roles as molecular chaperones and folding catalysts, suggesting their involvement in protein folding, quality control, and cellular homeostasis maintenance. Additionally, significant enrichment was observed in environmental adaptation pathways, which, combined with our previous expression data, indicates that *IbHSP90* genes participate in sweetpotato responses to abiotic stresses. Collectively, these findings confirm the functional conservation of *IbHSP90* in protein homeostasis and highlight its role in plant environmental adaptation. (**Supplementary Figure 4**).

Subcellular localization of HSP90 proteins varies considerably across plant species, reflecting functional divergence and adaptive specialization. For example, GmHsp90A2 from soybean localizes to both the cytoplasm and cell membrane, OsHSP90–1 and OsHSP90–4 from rice are distributed in the endoplasmic reticulum, and HbHSP90.1 from rubber tree is targeted to the nucleus (Huang et al., 2019; Liu et al., 2022; Wang et al., 2024). In the present study, transient expression of EGFP-tagged IbHSP90–2 in tobacco leaves showed that green fluorescence was concentrated in the nucleus and plasma membrane (**Figure 13**). This pattern suggests that IbHSP90–2 may act as a molecular chaperone in the cytoplasm and form functional regulatory complexes with target proteins within the nucleus. Yeast is a widely used model system for rapid preliminary characterization of gene function (Xiang et al., 2020). Heterologous expression of *IbHSP90–2* in yeast significantly reduced cold tolerance under low-temperature stress (**Figure 14**). Taken together with our expression profiles showing broad downregulation of IbHSP90 genes under cold treatment, these results support that IbHSP90–2 and closely related family members likely function as negative regulators in the sweetpotato cold stress response. The precise molecular and regulatory mechanisms underlying this repression, however, remain to be further investigated.

## 5 Conclusion

This study identified 41 *HSP90* family members in four *Ipomoea* species, characterized their conserved domains, analyzed intron-exon structures, and explored evolutionary relationships between sweetpotato and its three wild diploid relatives. Promoter analysis of *IbHSP90s* revealed 6 plant growth and development-associated, 137 abiotic stress-responsive, and 61 phytohormone-responsive cis-acting elements. We also assessed tissue-specific expression patterns of IbHSP90s, as well as their expression profiles induced by heat, cold, salt, drought stresses, and multiple phytohormones. Subcellular localization of GFP-tagged IbHSP90–2 in *N.benthamiana* leaves showed GFP signals localized to both the nucleus and plasma membrane. Functional analyses through heterologous expression reveal that IbHSP90s function as a negative regulator under cold stress. Future work will involve generating transgenic sweetpotato plants with both overexpression and RNAi-mediated silencing of IbHSP90–2 to functionally characterize its role in cold tolerance under field-relevant conditions.

## Data Availability

The original contributions presented in the study are included in the article/**Supplementary Material**. Further inquiries can be directed to the corresponding authors.

## References

[B1] Bailey T. L. Boden M. Buske F. A. Frith M. Grant C. E. Clementi L. . (2009). MEME SUITE: tools for motif discovery and searching. Nucleic Acids Res. 37, W202–W208. doi: 10.1093/nar/gkp335, PMID: 19458158 PMC2703892

[B2] Bettaieb I. Hamdi J. Bouktila D. (2020). Genome-wide analysis of HSP90 gene family in the Mediterranean olive (Olea europaea subsp. europaea) provides insight into structural patterns, evolution and functional diversity. Physiol. Mol. Biol. Plants 26, 2301–2318. doi: 10.1007/s12298-020-00888-x, PMID: 33268931 PMC7688888

[B3] Bittner A. Cieśla A. Gruden K. Lukan T. Mahmud S. Teige M. . (2022). Organelles and phytohormones: a network of interactions in plant stress responses. J. Exp. Bot. 73, 7165–7181. doi: 10.1093/jxb/erac384, PMID: 36169618 PMC9675595

[B4] Botër M. Amigues B. Peart J. Breuer C. Kadota Y. Casais C. . (2007). Structural and functional analysis of SGT1 reveals that its interaction with HSP90 is required for the accumulation of Rx, an R protein involved in plant immunity. Plant Cell 19, 3791–3804. doi: 10.1105/tpc.107.050427, PMID: 18032631 PMC2174866

[B5] Castellano M. M. Muñoz A. Okeke I. C. Novo-Uzal E. Toribio R. Mangano S. (2024). The role of the co-chaperone HOP in plant homeostasis during development and stress. J. Exp. Bot. 75, 4274–4286. doi: 10.1093/jxb/erae013, PMID: 38330220 PMC11263486

[B6] Chaudhary R. Baranwal V. K. Kumar R. Sircar D. Chauhan H. (2019). Genome-wide identification and expression analysis of Hsp70, Hsp90, and Hsp100 heat shock protein genes in barley under stress conditions and reproductive development. Funct. Integr. Genomics 19, 1007–1022. doi: 10.1007/s10142-019-00695-y, PMID: 31359217

[B7] Chen C. Chen H. Zhang Y. Thomas H. R. Frank M. H. He Y. . (2020). TBtools: an integrative toolkit developed for interactive analyses of big biological data. Mol. Plant 13, 1194–1202. doi: 10.1016/j.molp.2020.06.009, PMID: 32585190

[B8] Chen C. Wu Y. Li J. Wang X. Zeng Z. Xu J. . (2023). TBtools-II: A “one for all, all for one” bioinformatics platform for biological big-data mining. Mol. Plant 16, 1733–1742. doi: 10.1016/j.molp.2023.09.010, PMID: 37740491

[B9] Chen X. Kou M. Tang Z. Zhang A. Li H. Wei M. (2017). Responses of root physiological characteristics and yield of sweet potato to humic acid urea fertilizer. PloS One 12, e0189715. doi: 10.1371/journal.pone.0189715, PMID: 29253886 PMC5734739

[B10] Chou K. C. Shen H. B. (2010). Plant-mPLoc: a top-down strategy to augment the power for predicting plant protein subcellular localization. PloS One 5, e11335. doi: 10.1371/journal.pone.0011335, PMID: 20596258 PMC2893129

[B11] Ciura J. Kruk J. (2018). Phytohormones as targets for improving plant productivity and stress tolerance. J. Plant Physiol. 229, 32–40. doi: 10.1016/j.jplph.2018.06.013, PMID: 30031159

[B12] Driedonks N. Xu J. Peters J. L. Park S. Rieu I. (2015). Multi-level interactions between heat shock factors, heat shock proteins, and the redox system regulate acclimation to heat. Front. Plant Sci. 6. doi: 10.3389/fpls.2015.00999, PMID: 26635827 PMC4647109

[B13] Gasteiger E. Gattiker A. Hoogland C. Ivanyi I. Appel R. D. Bairoch A. (2003). ExPASy: The proteomics server for in-depth protein knowledge and analysis. Nucleic Acids Res. 31, 3784–3788. doi: 10.1093/nar/gkg563, PMID: 12824418 PMC168970

[B14] Gogoi K. Gogoi H. Borgohain M. Saikia R. Chikkaputtaiah C. Hiremath S. . (2024). The molecular dynamics between reactive oxygen species (ROS), reactive nitrogen species (RNS) and phytohormones in plant’s response to biotic stress. Plant Cell Rep. 43, 263. doi: 10.1007/s00299-024-03343-3, PMID: 39412663

[B15] Guo F. Lv M. Zhang J. Li J. (2024). Crosstalk between brassinosteroids and other phytohormones during plant development and stress adaptation. Plant Cell Physiol. 65, 1530–1543. doi: 10.1093/pcp/pcae047, PMID: 38727547

[B16] Hahn A. Bublak D. Schleiff E. Scharf K. D. (2011). Crosstalk between Hsp90 and Hsp70 chaperones and heat stress transcription factors in tomato. Plant Cell 23, 741–755. doi: 10.1105/tpc.110.076018, PMID: 21307284 PMC3077788

[B17] Hao Z. Y. Feng Q. Man X. Y. Qi D. Q. Qing Y. S. Yang Z. W. . (2025). Genome-wide identification and characterization of HSP90 family gene in cotton and their potential role in salt stress tolerance. Front. Plant Sci. 16. doi: 10.3389/fpls.2025.1574604, PMID: 40672555 PMC12263452

[B18] Hu M. Li Z. Lin X. Tang B. Xing M. Zhu H. (2024). Comparative analysis of the LEA gene family in seven Ipomoea species, focuses on sweet potato (Ipomoea batatas L.). BMC Plant Biol. 24, 1256. doi: 10.1186/s12870-024-05981-x, PMID: 39725899 PMC11670493

[B19] Huang Y. Xuan H. Yang C. Guo N. Wang H. Zhao J. . (2019). GmHsp90A2 is involved in soybean heat stress as a positive regulator. Plant Sci. 285, 26–33. doi: 10.1016/j.plantsci.2019.04.016, PMID: 31203891

[B20] Jia L. C. Yang Z. T. Shang L. L. He S. Z. Zhang H. Li X. . (2024). Genome-wide identification and expression analysis of the KNOX family and its diverse roles in response to growth and abiotic tolerance in sweet potato and its two diploid relatives. BMC Genomics 25, 572. doi: 10.1186/s12864-024-10470-4, PMID: 38844832 PMC11157901

[B21] Jogawat A. Yadav B. Chhaya N. Lakra Singh A. K. Narayan O. P. (2021). Crosstalk between phytohormones and secondary metabolites in the drought stress tolerance of crop plants: A review. Physiol. Plant 172, 1106–1132. doi: 10.1111/ppl.13328, PMID: 33421146

[B22] Johnson J. L. Brown C. (2009). Plasticity of the Hsp90 chaperone machine in divergent eukaryotic organisms. Cell Stress Chaperones. 14, 83–94. doi: 10.1007/s12192-008-0058-9, PMID: 18636345 PMC2673905

[B23] Lescot M. Déhais P. Thijs G. Marchal K. Moreau Y. Van de Peer Y. . (2002). PlantCARE, a database of plant cis-acting regulatory elements and a portal to tools for in silico analysis of promoter sequences. Nucleic Acids Res. 30, 325–327. doi: 10.1093/nar/30.1.325, PMID: 11752327 PMC99092

[B24] Letunic I. Bork P. (2021). Interactive Tree Of Life (iTOL) v5: an online tool for phylogenetic tree display and annotation. Nucleic Acids Res. 49, W293–w296. doi: 10.1093/nar/gkab301, PMID: 33885785 PMC8265157

[B25] Li W. Chen Y. Ye M. Wang D. Chen Q. (2020). Evolutionary history of the heat shock protein 90 (Hsp90) family of 43 plants and characterization of Hsp90s in Solanum tuberosum. Mol. Biol. Rep. 47, 6679–6691. doi: 10.1007/s11033-020-05722-x, PMID: 32780253

[B26] Liu H. Zhang Y. Li X. Zhao L. Ma X. He F. . (2025). Genome-wide identification and analysis of the abiotic stress responsiveness of the heat shock protein 90 gene family in Medicago sativa L. BMC Plant Biol. 25, 1498. doi: 10.1186/s12870-025-07520-8, PMID: 41184778 PMC12581226

[B27] Liu M. Wang L. Ke Y. Xian X. Wang J. Wang M. . (2022). Identification of HbHSP90 gene family and characterization HbHSP90.1 as a candidate gene for stress response in rubber tree. Gene 827, 146475. doi: 10.1016/j.gene.2022.146475, PMID: 35378248

[B28] Liu Y. Chen Q. Zhou M. Yang X. Yang C. Jiao C. (2020). Sweet potato study in China: Stress response mechanisms, molecular breeding, and productivity. J. Plant Physiol. 254, 153283. doi: 10.1016/j.jplph.2020.153283, PMID: 32961476

[B29] Meng X. Dong T. Li Z. Zhu M. (2024). First systematic review of the last 30 years of research on sweetpotato: elucidating the frontiers and hotspots. Front. Plant Sci. 15. doi: 10.3389/fpls.2024.1428975, PMID: 39036362 PMC11258629

[B30] Milioni D. Hatzopoulos P. (1997). Genomic organization of hsp90 gene family in Arabidopsis. Plant Mol. Biol. 35, 955–961. doi: 10.1023/a:1005874521528, PMID: 9426614

[B31] Mistry J. Chuguransky S. Williams L. Qureshi M. Salazar G. A. Sonnhammer E. L. L. . (2021). Pfam: The protein families database in 2021. Nucleic Acids Res. 49, D412–d419. doi: 10.1093/nar/gkaa913, PMID: 33125078 PMC7779014

[B32] Moraes M. B. F. Daúde M. M. De Oliveira K. K. P. Gonçalves R. C. Ságio S. A. Lima A. A. . (2025). Evaluation and validation of reference genes for RT-qPCR normalization in different sweet potato tissues. Sci. Rep. 15, 39899. doi: 10.1038/s41598-025-22650-7, PMID: 41238620 PMC12618874

[B33] Morimoto R. I. (1998). Regulation of the heat shock transcriptional response: cross talk between a family of heat shock factors, molecular chaperones, and negative regulators. Genes Dev. 12, 3788–3796. doi: 10.1101/gad.12.24.3788, PMID: 9869631

[B34] Peng J. Liu S. Wu J. Liu T. Liu B. Xiong Y. . (2024). Genome-wide analysis of the oat (Avena sativa) HSP90 gene family reveals its identification, evolution, and response to abiotic stress. Int. J. Mol. Sci. 25, 2305. doi: 10.3390/ijms25042305, PMID: 38396983 PMC10889330

[B35] Peng J. L. Xu H. N. Yang W. L. Li X. (2025). Genome-wide identification and expression analysis of the ferritin family in sweetpotato and its two diploid relatives. BMC Plant Biol. 25, 765. doi: 10.1186/s12870-025-06732-2, PMID: 40474077 PMC12139147

[B36] Picard D. (2002). Heat-shock protein 90, a chaperone for folding and regulation. Cell Mol. Life Sci. 59, 1640–1648. doi: 10.1007/pl00012491, PMID: 12475174 PMC11337538

[B37] Pratt W. B. Toft D. O. (2003). Regulation of signaling protein function and trafficking by the hsp90/hsp70-based chaperone machinery. Exp. Biol. Med. (Maywood). 228, 111–133. doi: 10.1177/153537020322800201, PMID: 12563018

[B38] Prodromou C. Pearl L. H. (2003). Structure and functional relationships of Hsp90. Curr. Cancer Drug Targets 3, 301–323. doi: 10.2174/1568009033481877, PMID: 14529383

[B39] Ren S. Wang H. Jiao Y. Wang Y. (2026). Heat shock protein modulates cell expansion via ROS homeostasis. New Phytol. doi: 10.1111/nph.70970, PMID: 41645536

[B40] Sadura I. Libik-Konieczny M. Jurczyk B. Gruszka D. Janeczko A. (2020). HSP transcript and protein accumulation in brassinosteroid barley mutants acclimated to low and high temperatures. Int. J. Mol. Sci. 21, 1889. doi: 10.3390/ijms21051889, PMID: 32164259 PMC7084868

[B41] Sajad S. Jiang S. Anwar M. Dai Q. Luo Y. Hassan M. A. . (2022). Genome-Wide Study of Hsp90 Gene Family in Cabbage (Brassica oleracea var. capitata L.) and Their Imperative Roles in Response to Cold Stress. Front. Plant Sci. 13. doi: 10.3389/fpls.2022.908511, PMID: 35812899 PMC9258498

[B42] Samakovli D. Roka L. Dimopoulou A. Plitsi P. K. Žukauskait A. Georgopoulou P. . (2021). HSP90 affects root growth in Arabidopsis by regulating the polar distribution of PIN1. New Phytol. 231, 1814–1831. doi: 10.1111/nph.17528, PMID: 34086995

[B43] Sangster T. A. Queitsch C. (2005). The HSP90 chaperone complex, an emerging force in plant development and phenotypic plasticity. Curr. Opin. Plant Biol. 8, 86–92. doi: 10.1016/j.pbi.2004.11.012, PMID: 15653405

[B44] Song W. Li C. Kou M. Li C. Gao G. Cai T. . (2024). Different regions and environments have critical roles on yield, main quality and industrialization of an industrial purple-fleshed sweetpotato (Ipomoea batatas L. (Lam.)) “Xuzishu8. Heliyon 10, e25328. doi: 10.1016/j.heliyon.2024.e25328, PMID: 38390079 PMC10881541

[B45] Song Z. Pan F. Yang C. Jia H. Jiang H. He F. . (2019). Genome-wide identification and expression analysis of HSP90 gene family in Nicotiana tabacum. BMC Genet. 20, 35. doi: 10.1186/s12863-019-0738-8, PMID: 30890142 PMC6423791

[B46] Song H. Zhao R. Fan P. Wang X. Chen X. Li Y. (2009). Overexpression of AtHsp90.2, AtHsp90.5 and AtHsp90.7 in Arabidopsis thaliana enhances plant sensitivity to salt and drought stresses. Planta 229, 955–964. doi: 10.1007/s00425-008-0886-y, PMID: 19148673

[B47] Stachurska J. Sadura I. Jurczyk B. Rudolphi-Szydło E. Dyba B. Pociecha E. . (2024). Cold acclimation and deacclimation of winter oilseed rape, with special attention being paid to the role of brassinosteroids. Int. J. Mol. Sci. 25. doi: 10.3390/ijms25116010, PMID: 38892204 PMC11172585

[B48] Sun H. Mu T. Xi L. Zhang M. Chen J. (2014). Sweet potato (Ipomoea batatas L.) leaves as nutritional and functional foods. Food Chem. 156, 380–389. doi: 10.1016/j.foodchem.2014.01.079, PMID: 24629984

[B49] Tamura K. Stecher G. Kumar S. (2021). MEGA11: molecular evolutionary genetics analysis version 11. Mol. Biol. Evol. 38, 3022–3027. doi: 10.1093/molbev/msab120, PMID: 33892491 PMC8233496

[B50] Thirumalaikumar V. P. Gorka M. Schulz K. Masclaux-Daubresse C. Sampathkumar A. Skirycz A. . (2021). Selective autophagy regulates heat stress memory in Arabidopsis by NBR1-mediated targeting of HSP90.1 and ROF1. Autophagy 17, 2184–2199. doi: 10.1080/15548627.2020.1820778, PMID: 32967551 PMC8496721

[B51] Von Mering C. Jensen L. J. Snel B. Hooper S. D. Krupp M. Foglierini M. . (2005). STRING: known and predicted protein-protein associations, integrated and transferred across organisms. Nucleic Acids Res. 33, D433–D437. doi: 10.1093/nar/gki005, PMID: 15608232 PMC539959

[B52] Wang H. Charagh S. Dong N. Lu F. Wang Y. Cao R. . (2024). Genome-wide analysis of heat shock protein family and identification of their functions in rice quality and yield. Int. J. Mol. Sci. 25, 11931. doi: 10.3390/ijms252211931, PMID: 39596001 PMC11593806

[B53] Wang H. Dong Z. Chen J. Wang M. Ding Y. Xue Q. . (2022). Genome-wide identification and expression analysis of the Hsp20, Hsp70 and Hsp90 gene family in Dendrobium officinale. Front. Plant Sci. 13. doi: 10.3389/fpls.2022.979801, PMID: 36035705 PMC9399769

[B54] Wang X. R. Wang C. Ban F. X. Zhu D. T. Liu S. S. Wang X. W. (2019). Genome-wide identification and characterization of HSP gene superfamily in whitefly (Bemisia tabaci) and expression profiling analysis under temperature stress. Insect Sci. 26, 44–57. doi: 10.1111/1744-7917.12505, PMID: 28714602

[B55] Wei Y. Liu W. Hu W. Yan Y. Shi H. (2020). The chaperone MeHSP90 recruits MeWRKY20 and MeCatalase1 to regulate drought stress resistance in cassava. New Phytol. 226, 476–491. doi: 10.1111/nph.16346, PMID: 31782811

[B56] Wei Y. Zhu B. Zhang Y. Ma G. Wu J. Tang L. . (2024). CPK1-HSP90 phosphorylation and effector XopC2-HSP90 interaction underpin the antagonism during cassava defense-pathogen infection. New Phytol. 242, 2734–2745. doi: 10.1111/nph.19739, PMID: 38581188

[B57] Wu S. Lau K. H. Cao Q. Hamilton J. P. Sun H. Zhou C. . (2018). Genome sequences of two diploid wild relatives of cultivated sweetpotato reveal targets for genetic improvement. Nat. Commun. 9, 4580. doi: 10.1038/s41467-018-06983-8, PMID: 30389915 PMC6214957

[B58] Wu S. Sun H. Zhao X. Hamilton J. P. Mollinari M. Gesteira G. S. . (2025). Phased chromosome-level assembly provides insight into the genome architecture of hexaploid sweetpotato. Nat. Plants 11, 1951–1959. doi: 10.1038/s41477-025-02079-6, PMID: 40781486

[B59] Xiang D. J. Man L. L. Cao S. Liu P. Li Z. G. Wang X. D. (2020). Heterologous expression of an Agropyron cristatum SnRK2 protein kinase gene (AcSnRK2.11) increases freezing tolerance in transgenic yeast and tobacco. 3. Biotech. 10, 209. doi: 10.1007/s13205-020-02203-7, PMID: 32351867 PMC7181469

[B60] Xiao D. Jiang Y. Wang Z. Li X. Li H. Tang S. . (2025). Genome-wide identification and expression analysis of the HSP90 gene family in relation to developmental and abiotic stress in ginger (Zingiber officinale roscoe). Plants (Basel). 14, 1660. doi: 10.3390/plants14111660, PMID: 40508332 PMC12157278

[B61] Xiao S. Wang Y. Zhou Z. Zhao L. Zhao L. Gao B. . (2025). Xiaoshu, a simple genetic model system for sweetpotato (Ipomoea batatas (L.) Lam.). Plant Biotechnol. J. 23, 674–676. doi: 10.1111/pbi.14528, PMID: 39620264 PMC11772304

[B62] Xu J. Liu S. Ren Y. You Y. Wang Z. Zhang Y. . (2024). Genome-wide identification of HSP90 gene family in Rosa chinensis and its response to salt and drought stresses. 3. Biotech. 14, 204. doi: 10.1007/s13205-024-04052-0, PMID: 39161880 PMC11330952

[B63] Xue P. Sun Y. Hu D. Zhang J. Wan X. (2023). Genome-wide characterization of DcHsp90 gene family in carnation (Dianthus caryophyllus L.) and functional analysis of DcHsp90–6 in heat tolerance. Protoplasma 260, 807–819. doi: 10.1007/s00709-022-01815-5, PMID: 36264387

[B64] Yadav P. K. Gupta N. Verma V. Gupta A. K. (2021). Overexpression of SlHSP90.2 leads to altered root biomass and architecture in tomato (Solanum lycopersicum). Physiol. Mol. Biol. Plants 27, 713–725. doi: 10.1007/s12298-021-00976-6, PMID: 33967458 PMC8055811

[B65] Yan M. Li M. Wang Y. Wang X. Moeinzadeh M. H. Quispe-Huamanquispe D. G. . (2024). Haplotype-based phylogenetic analysis and population genomics uncover the origin and domestication of sweetpotato. Mol. Plant 17, 277–296. doi: 10.1016/j.molp.2023.12.019, PMID: 38155570

[B66] Yan M. Nie H. Wang Y. Wang X. Jarret R. Zhao J. . (2022). Exploring and exploiting genetics and genomics for sweetpotato improvement: Status and perspectives. Plant Commun. 3, 100332. doi: 10.1016/j.xplc.2022.100332, PMID: 35643086 PMC9482988

[B67] Yang J. Moeinzadeh M. H. Kuhl H. Helmuth J. Xiao P. Haas S. . (2017). Haplotype-resolved sweet potato genome traces back its hexaploidization history. Nat. Plants 3, 696–703. doi: 10.1038/s41477-017-0002-z, PMID: 28827752

[B68] Yuan C. Li C. Zhao X. Yan C. Wang J. Mou Y. . (2021). Genome-wide identification and characterization of HSP90-RAR1-SGT1-complex members from arachis genomes and their responses to biotic and abiotic stresses. Front. Genet. 12. doi: 10.3389/fgene.2021.689669, PMID: 34512718 PMC8430224

[B69] Zhang J. Li J. Liu B. Zhang L. Chen J. Lu M. (2013). Genome-wide analysis of the Populus Hsp90 gene family reveals differential expression patterns, localization, and heat stress responses. BMC Genomics 14, 532. doi: 10.1186/1471-2164-14-532, PMID: 23915275 PMC3750472

