# Supplementary material for: Genome-wide comparative analysis of the HSP90 gene family in four Ipomoea species and functional insights into the potentiality of IbHSP90–2 in low temperature tolerance
Source: Front Plant Sci. 2026 Mar 6;17:1791008. doi: 10.3389/fpls.2026.1791008 (PMC13003396; doi:10.3389/fpls.2026.1791008)
Supplement: Supplementary file 2 [file DataSheet1.docx]

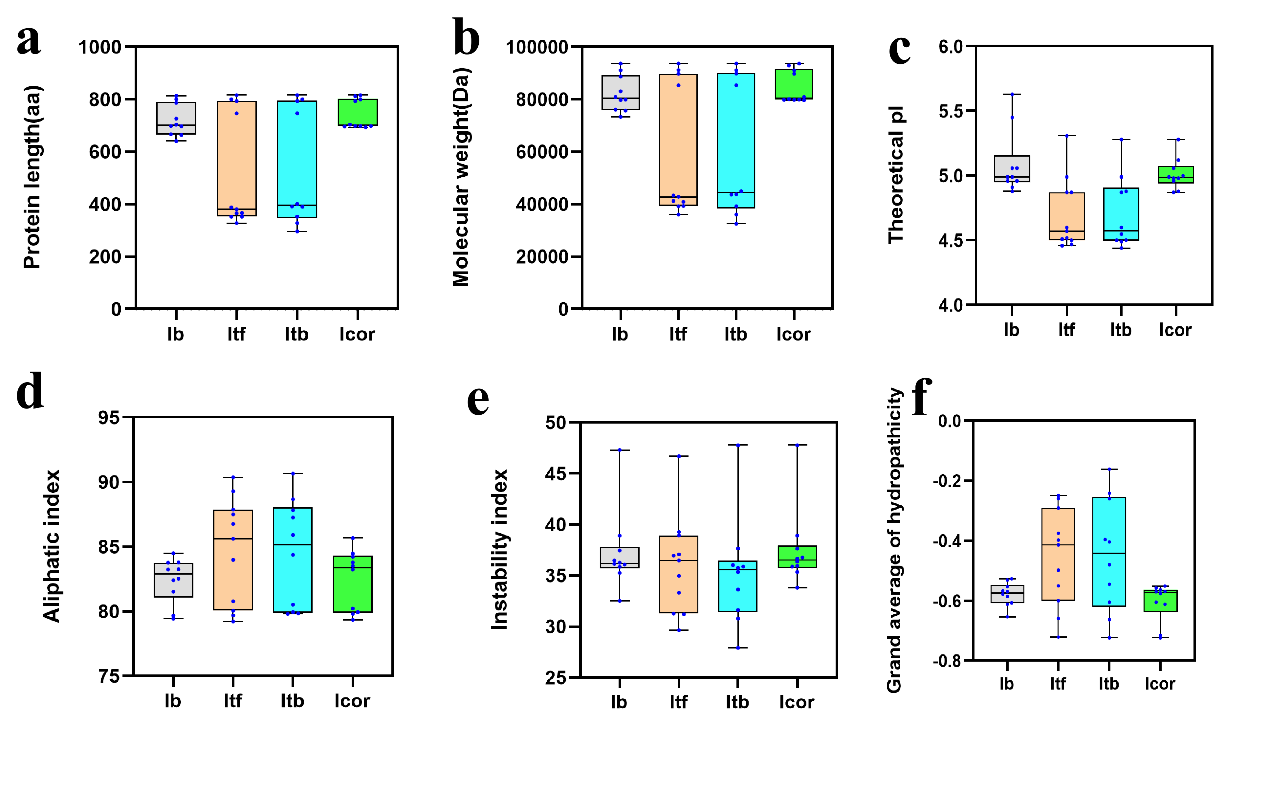


**FigS1** The physicochemical properties of HSP90 proteins in four Ipomoea species(Ib: *I.batatas*, Itf: *I.trifida*, Itb: *I.triloba*, I.cor: *I.cordatotriloba*), including:Protein length(**a**), Molecular weight(**b**), Theoretical pI(**c**), Aliphatic index(**d**), Instability index(**e**), Grand average of hydropathicity (**f**).


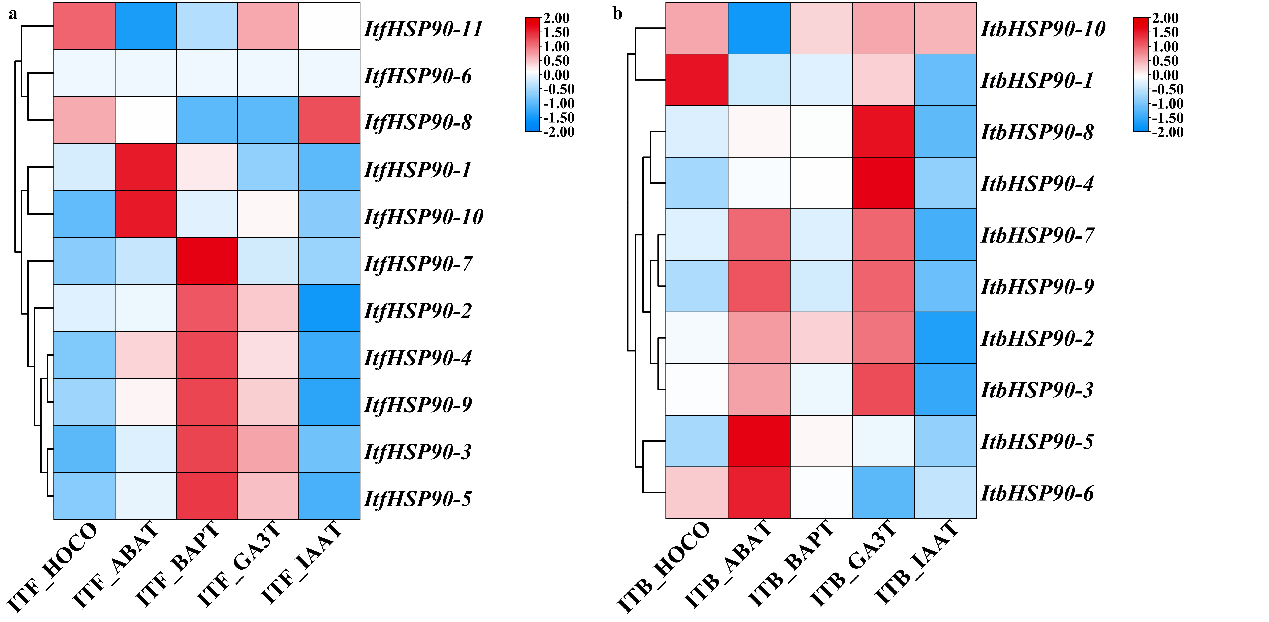


**FigS2** Expression analysis of *HSP90s* in *I. trifida* and *I. triloba* under hormonal treatment as determined by RNA−seq.(a) Expression analysis of *ItfHSP90s* in *I. trifida* under hormonal treatment. ITF_HOCO: *Ipomoea trifida* hormone control experiment. ITF_ABAT: *Ipomoea trifida* abscisic acid 50 μM hormone stress experiment.ITF_BAPT: *Ipomoea trifida* 6-benzylaminopurine 10 μM hormone stress experiment. ITF_GA3T: *Ipomoea trifida* gibberellic acid 50 μM hormone stress experiment. ITF_IAAT: *Ipomoea trifida* Indole-3-acetic acid 10 μM hormone stress experiment (b) Expression analysis of *ItbHSP90s* in *I. triloba* under hormonal treatment. ITB_HOCO: *Ipomoea triloba* hormone control experiment. ITB_ABAT: *Ipomoea triloba* abscisic acid 50 μM hormone stress experiment. ITB_BAPT: *Ipomoea triloba* 6-benzylaminopurine 10 μM hormone stress experiment. ITB_GA3T: *Ipomoea triloba* gibberellic acid 50 μM hormone stress experiment. ITB_IAAT: Ipomoea triloba Indole-3-acetic acid 10 μM hormone stress experiment.


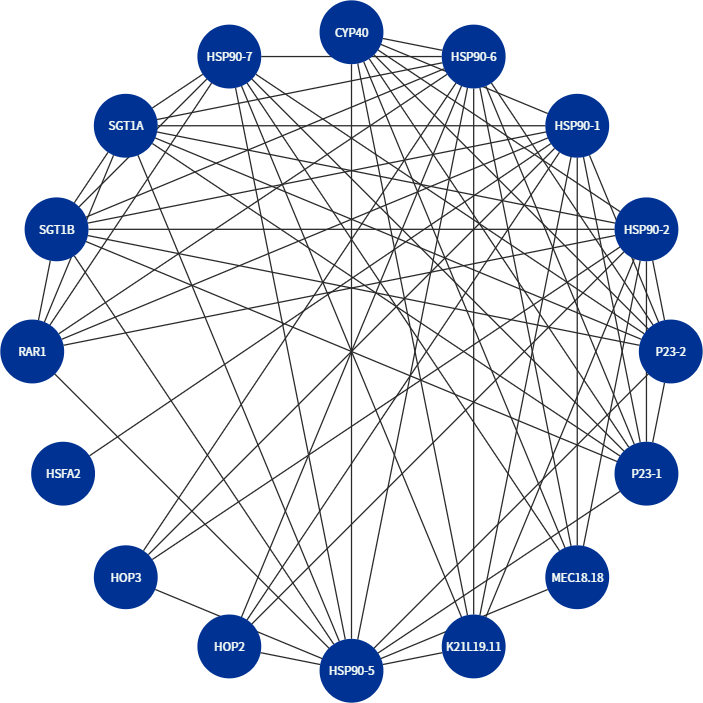


**FigS3** PPI of HSP90 proteins was drawn using Cytoscape and String's online database.


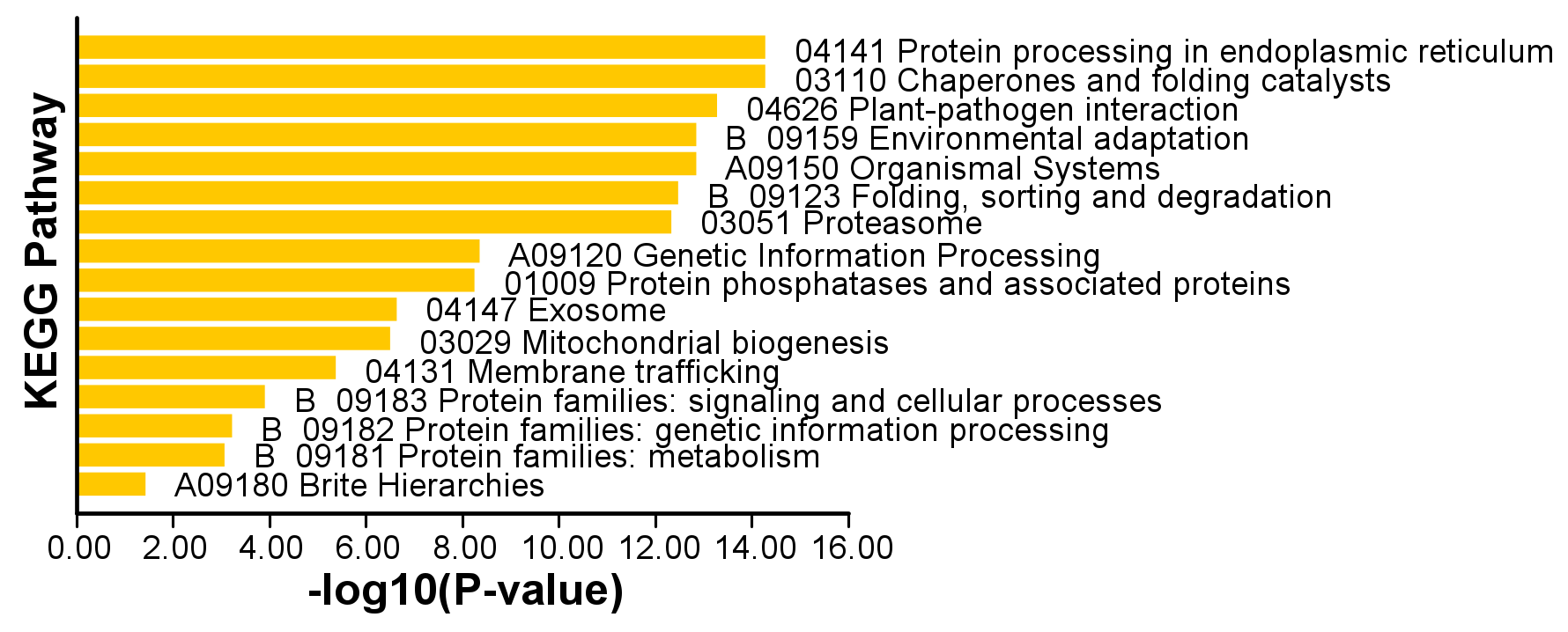


**FigS4** KEGG functional annotation analysis.
